# Tropical cyclone impacts on seagrass-associated fishes in a temperate-subtropical estuary

**DOI:** 10.1371/journal.pone.0273556

**Published:** 2022-10-13

**Authors:** Y. Stacy Zhang, Savannah H. Swinea, Grace Roskar, Stacy N. Trackenberg, Rachel K. Gittman, Jessie C. Jarvis, W. Judson Kenworthy, Lauren A. Yeager, F. Joel Fodrie

**Affiliations:** 1 Institute of Marine Sciences and Department of Earth, Marine and Environmental Sciences, University of North Carolina at Chapel Hill, Morehead City, North Carolina, United States of America; 2 Department of Marine and Environmental Sciences, Northeastern University, Marine Science Center, Nahant, Massachusetts, United States of America; 3 North Carolina Coastal Reserve and National Estuarine Research Reserve, Beaufort, North Carolina, United States of America; 4 Department of Biology, East Carolina University, Greenville, North Carolina, United States of America; 5 Coastal Studies Institute, East Carolina University, Greenville, North Carolina, United States of America; 6 Department of Biology and Marine Biology, University of North Carolina Wilmington, Wilmington, NC, United States of America; Commonwealth Scientific and Industrial Research Organisation, AUSTRALIA

## Abstract

Major storms can alter coastal ecosystems in several direct and indirect ways including habitat destruction, stormwater-related water quality degradation, and organism mortality. From 2010–2020, ten tropical cyclones impacted coastal North Carolina, providing an opportunity to explore ecosystem responses across multiple storms. Using monthly trawl and contemporaneous seagrass surveys conducted in Back Sound, NC, we evaluated how cyclones may affect the nursery role of shallow-water biogenic habitats by examining seagrass-associated fish responses within a temperate-subtropical estuary. We employed a general before-after-control-impact approach using trawls conducted prior (before) and subsequent (after) to storm arrival and years either without (control) or with (impact) storms. We examined whether effects were apparent over short (within ~three weeks of impact) and seasonal (May-October) timescales, as well as if the magnitude of storm-related shifts varied as a function of storm intensity. Our findings suggest that the ability of these shallow-water habitats to support juvenile fishes was not dramatically altered by hurricanes. The resilience exhibited by fishes was likely underpinned by the relative persistence of the seagrass habitat, which appeared principally undamaged by storms based upon review of available–albeit limited seagrass surveys. Increasing cyclone intensity, however, was correlated with greater declines in catch and may potentially underlie the emigration and return rate of fish after cyclones. Whether estuarine fishes will continue to be resilient to acute storm impacts despite chronic environmental degradation and predicted increases major tropical cyclone frequency and intensity remains a pressing question.

## Introduction

Pulse disturbances are defined as strong, short-term perturbations that cause a sudden change in ecosystem structure from which the system can recover when stress has subsided. In contrast, press disturbances are typified as a continuous perturbation or stressor that causes a permanent change in ecosystem structure [1, 2]. Tropical cyclones (e.g., hurricanes and typhoons) are typically characterized as extreme pulse disturbances, and current forecasts predict an increase in major tropical cyclone frequency, intensity, and geographical scope as global climate change continues [3–6]. Combined, these ramping forces have the potential to de-stabilize coastal ecosystems and lead to long-term changes in community structure [7]. Thus, understanding the capacity for resistance and resilience within estuarine ecosystems, particularly seagrass meadows that serve as fish nursery habitats, will be critical for forecasting if and how coastal systems shift under these changing climate scenarios.

Estuarine ecosystems are strongly impacted by a variety of interlinked biotic, geophysical, and socioecological processes from both terrestrial and marine sources to form a highly dynamic environment [8]. Water temperature and salinity are spatially and temporally heterogeneous within an estuary and can change rapidly depending upon depth, tides, weather, and other factors that further affect dissolved oxygen content, nutrient fluxes, and water quality [9]. Seasonal patterns of river discharge and warming add an additional layer of biogeochemical complexity and can dictate productivity across trophic levels [10]. Because of these, estuarine flora and fauna are often adapted to withstand high variability in physical conditions [11, 12]. Still, extreme environmental conditions can stress and degrade estuarine foundation species such as seagrasses, salt marshes, and oysters along with the organisms that rely upon them [13–16]. Physical stress from strong winds, waves, and storm surge associated with hurricanes can scour or erode coastal habitats and flood up-estuary mesohaline areas with sea water, while inland rains can inundate coastal estuaries with high volumes of stormwater runoff laden with terrestrial exports that shift physiochemical regimes over hourly-to-monthly scales [17–19]. Combined, large influxes of either fresh- or saltwater can displace organisms from preferred estuarine habitats and cause localized mortality and increased morbidity that shifts community composition towards more freshwater or marine communities, depending on location within the watershed [20–23].

While some fishes can detect environmental cues associated with approaching storms and respond by temporarily seeking refugia [23–27], cyclone-induced biogeochemical shifts can have disproportionate impacts on juvenile fishes by displacing prey, decreasing body condition, and increasing disease prevalence and morbidity [28, 29]. Studies that have examined the impacts of hurricanes on fish communities in shallow coastal ecosystems including estuaries, coral reefs, seagrasses, mangroves, and other nearshore ocean habitats have typically compared conditions before and after singular storm events (Table 1). These studies have found disparate responses: 27% documented abundance decreases, 18% found abundance increases, 14% recorded shifts in species composition but no change in overall abundance, and 41% found no detectable effect within 6 months of hurricane strike (6, 4, 3, and 9 studies, respectively). Over longer time frames (> 6 months after storm), only 3 of 15 studies observed changes in fish abundance, with two documenting post-storm increases [30] and one finding post-storm declines [28]. Notably, the majority were case studies focused on a single hurricane event (16/22) and only seven examined seagrass-associated fishes.

**Table 1:** Summary table of studies (n = 22) examining change in fish abundance before and after cyclone.

Study	Location	Habitat	Year	Saff Simp*	< 6 mo	> 6 mo
Adams 2001	USVI (Carib. Oce)	Coral Reef	1995	2	No effect	
Adams & Ebersole 2004	USVI (Carib. Oce)	Coral Reef, Seagrass	1999	4	No effect	No effect
Anton et al. 2009	Alabama (USA)	Seagrass	2005	4	No effect	No effect
Bortone 1976	Florida (USA)	Jetty, Estuary	1975	3	Decline	
Bouchon et al. 1994	Guadeloupe (Carib. Oce.)	Mangrove, Coral Reef, Seagrass	1989	4	Decline	
Burkholder et al. 2004	North Carolina (USA)	Estuary	1996, 1999	3,TS*, 2, 1	Decline	No effect
Cheal et al. 2002	Queensland (AUS)	Coral Reef	1999	2	No effect	No effect
Davis & Laird 1976	Virginia (USA)	Estuary, Seagrass	1972	TS	No effect	
Dolloff et al. 1994	North Carolina (USA)	Streams and rivers	1989	4	No effect	No effect
Fenner 1991	Quintana Roo (MEX)	Coral Reef	1988	5	No effect	No effect
Fitzsimmons & Nishimoto 1995	Hawaii (Pac. Oce)	Streams and rivers	1992	4	Decline	No effect
Greenwood et al. 2006	Florida (USA)	Seagrass	2004	4, 2, 3, 3	No effect	No effect
Lassig 1983	Queensland (AUS)	Coral Reef	1981	TS	Assemblage change	
Letourneur et al. 1993	Reunion Island (Ind. Oce.)	Coral Reef	1989	2	Increase	Increase
Locascio & Mann, 2005	Florida (USA)	Estuary, Seagrass	2004	4	Increase	No effect
Paerl et al. 2001	North Carolina (USA)	Estuary, River	1999	TS, 2, 1	Decline	Decline
Paperno et al. 2006	Florida (USA)	Seagrass	2004	2, 3	Decline	No effect
Springer & McErlean 1962	Florida (USA)	Coral Reef	1960	4	No effect	
Stevens et al. 2006	Florida (USA)	Estuary, Seagrass	2004	4	Assemblage change	No effect
Switzer et al. 2006	Florida (USA)	Estuary	2004	2,3	Assemblage change	No effect
Yu et al. 2013	South China Sea (CHN)	Marginal Sea	2009, 2009	TS, 1	Increase	Increase
Yu et al 2014	South China Sea (CHN)	Marginal Sea	2010, 2012, 2012	1, 4, 1	Increase	Increase

Literature review of studies that examine fish abundances before and after hurricanes in coral reef, seagrass, estuary, and rocky bottom habitats. Effects were separated into responses that were observed within 6 months of storm passage and those observed after 6 months.

*Saff Simp = Saffir Simpson wind scale category, TS = tropical storm. Citations listed in S1 Appendix.

Estuarine fish communities in the temperate-subtropical transition zone of the western Atlantic (~30-35°N latitude) are comprised in significant part by species that ingress as larvae and spend much of their juvenile life stages in estuaries before migrating offshore as adults [31]. Seagrass meadows in this region are recognized as nursery habitats for their ability to enhance juvenile fish growth and survival [32]. It is hypothesized that seagrasses may be somewhat protected from tropical cyclone wave scour due to storm surge submergence, but increased turbidity, phytoplankton blooms, and sediment deposition post-storm can cause light limitation and subsequent mortality [33]. Studies of hurricane impacts on seagrasses have documented both no effect [34–38] as well as declines in productivity and extent [33, 39–44]. Meadows may recover more quickly if dominated by weedy, fast-growing seagrass species [43, 45], but the amount of time between disturbances may play the deciding role in overall ecosystem trajectory [46]. Long-term data on seagrass meadow cover, extent, and health prior to and post-hurricanes can help to elucidate changes in seagrass incurred from hurricane damage from covariates such as seasonal and interannual variation in productivity and how this relates to nursery habitat provisioning.

Our objective was to determine whether hurricanes affected seagrass and seagrass associated fishes and whether changes were correlated with tropical cyclone intensity. Specifically, we employed before-after-control-impact (BACI) frameworks to determine whether seagrass habitat, seagrass-associated fish catch, species richness, and community structure were impacted by hurricanes (winds ≥64 knots), with separate analyses to examine the effect of cyclones that made landfall during early (June-July), peak (August-September), and late (October-November) Atlantic basin hurricane season. We then examined whether increasing cyclone intensity was correlated with greater change in catch and richness for all tropical storms (winds ≥34 knots) and hurricanes to impact our area. Finally, to examine the potential longevity of responses, we examined whether storm impacts were evident over short (~three weeks) and/or longer (seasonal) timescales. We hypothesized that habitat persistence would underlie fish community dynamics [47–49]; thus, if local seagrass meadows were largely unaffected by tropical cyclones, fish communities would also be stable or resilient.

## Methods

### Tropical cyclones and surveys

**Fig 1:**
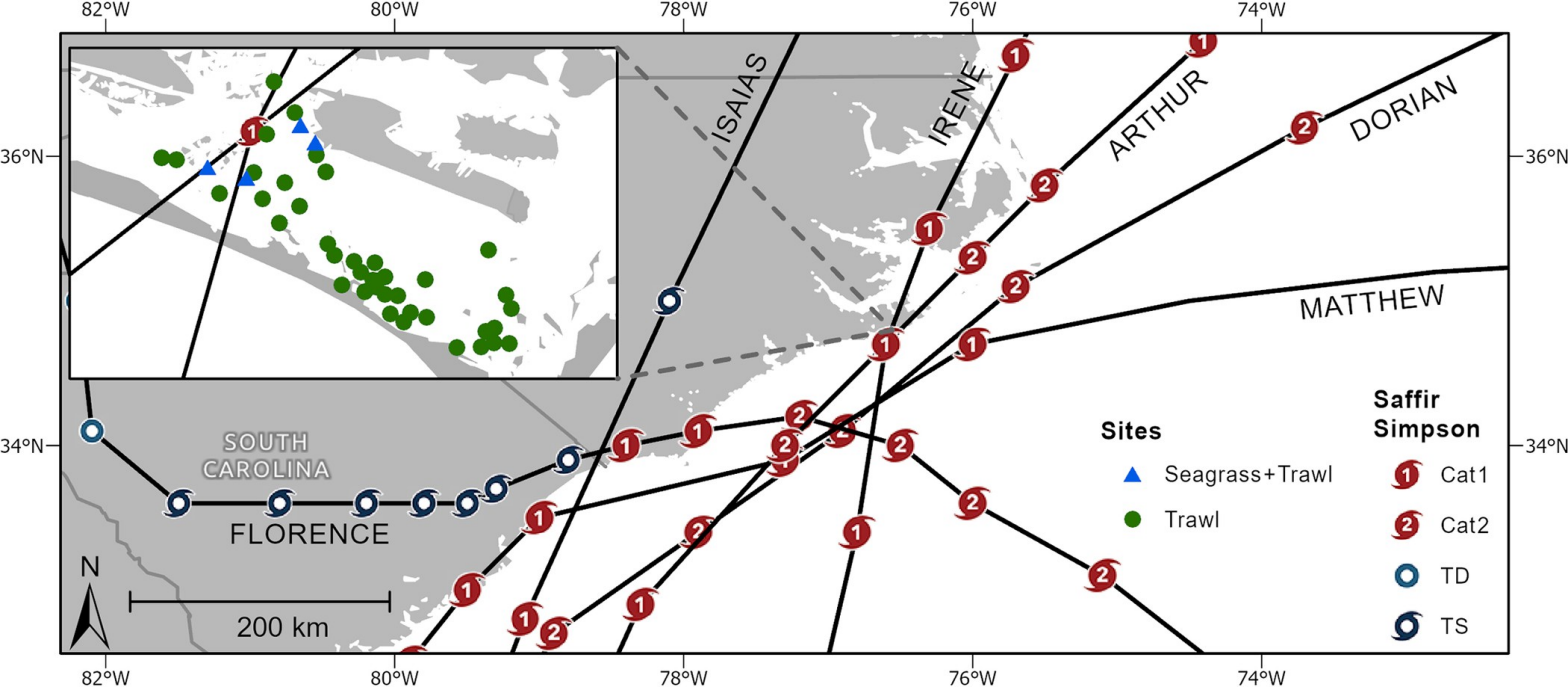
Major hurricane tracks over coastal North Carolina from 2010–2020. Tracks are based on best track data from HURDAT2 [54]. Cyclone symbology represents hurricane location and Saffir Simpson category at 6-hour intervals (TD = tropical depression, TS = tropical storm). Inset map depicts study area with green circles indicating trawl survey locations and blue triangles indicating seagrass survey locations. Map sources: Esri, HERE, Garmin, SafeGraph, Meti/NASA, USGS, EPA, NPS, USDA.

In the Atlantic basin, hurricane season occurs from June 1-Nov 30, with greatest activity occurring between mid-August to mid-October [50]. Our study focuses on the impact of tropical cyclones on seagrass meadows and seagrass-associated fishes within a temperate-subtropical embayment—Back Sound, North Carolina, USA (34° N to 34°39′ N, 76°37′ W to 76°31′ W)—that is among the most frequently cyclone-impacted locations within the continental United States. Back Sound is a shallow (mean depth ~2 m), lagoonal ecosystem enclosed by barrier islands to the south and east (Fig 1). Since 2010, 10 named storms have affected the study area with tropical storm force winds and above (≥34 knots & Table 2). Of these, six made landfall along or struck (came within 65 nautical miles, Fig 1) the study area with at least Saffir Simpson Category 1 (≥64 knot) winds: Hurricanes Irene (8/27/2011), Arthur (7/4/2014), Matthew (10/9/2016), Florence (9/14/2018), Dorian (9/6/2019), and Isaias (8/4/2020). These six hurricanes caused a combined ~30 billion dollars (USD) in storm-related damages to commercial, residential, and agricultural infrastructure within the state [51], with Hurricanes Matthew and Florence also producing 100–1000 year flood events [19]. Rainfall anomalies used in analyses were calculated as the difference between observed year-to-date rainfall and the 30-year (1991–2020) year-to-date normals for NWS Station 315830 [52, 53].

**Table 2:** North Carolina tropical cyclone and control year metrics 2010–2020.

Year	Name/ Treatment	Impact Date	ACE-NC	Winds (kt)	Gusts (kt)	Storm Surge (m)	Rainfall (cm)	Analyses
2010	TS Earl	3-Sep	2.59	25.27	30.71	0.17	1.25	Intensity
2011	Irene	27-Aug	5.09	33.82	46.07	0.88	22.23	mBACI, Comm, Intensity
2012	TS Beryl	30-May	1.12	26.24	31.49	0.14	7.34	Reg
2013	No Storm	14-Sep						mBACI, Comm
2014	Arthur	4-Jul	5.47	46.85	60.26	0.73	5.13	BACI
2015	No Storm	4-Jul, 6-Sep						mBACI, Comm BACI (v. Arthur)
2016	TS Hermine	3-Sep	1.27	25.66	34.41	0.44	1.65	Intensity (short-term)
2016	Matthew	9-Oct	4.23	38.88	57.15	0.64	3.33	BACI
2017	No Storm	8-Aug, 9-Oct						mBACI, Comm BACI (v. Matthew)
2018	Florence	14-Sep	12.44	51.32	71.53	1.45	29.19	BACI, Comm, Intensity
2018	Michael	11-Oct	2.63	34.76	46.51	0.27	0.91	Int (short-term)
2019	Dorian	6-Sep	3.79	--	73.86^♠^	0.63	14.30	mBACI, Comm, Intensity
2020	Isaias	4-Aug	1.59	42.36	52.28	0.32	3.40	mBACI, Comm, Intensity

Characteristics of tropical cyclones and no-storm years used in the listed analyses. Impact date represents date of first arrival of tropical storm force winds. For no storm years/treatments, dummy impact dates were assigned in order to bisect data into time periods and chosen to generally align with observed stormfall dates. ACE-NC = Accumulated Cyclone Energy calculated for the span of time in which the cyclone directly impacted North Carolina. TS = tropical storm as defined by the Saffir Simpson wind scale. Unless otherwise stated, analyses examined both short-term and seasonal time frames. mBACI = multiple Before-After-Control-Impact analysis of Hurricanes Irene, Florence, Dorian, and Isaias, BACI = individual comparison of either Hurricane Matthew or Hurricane Arthur against a single no-storm year, Comm = community structure analyses, Intensity = tobit or linear regression of change versus ACE-NC of all cyclones of tropical storm strength or greater.

Water levels, winds, and gusts were obtained from National Oceanic and Atmospheric Administration (NOAA) Station 8656483: Beaufort, Duke Marine Lab, North Carolina, USA (34°42’58"N 76°40’15"W). This station records 6 min. intervals for water levels & wind speeds; maximum recorded values are reported. Storm surge is calculated as the difference between predicted and measured tide. Rainfall data obtained from NOAA National Weather Service (NWS) Station 315830 in Morehead City, North Carolina, USA (34°42’58"N 76°40’15"W) (NOAA n.d., accessed 3 Feb 2021).

^♠^ Wind speeds were not available from the Station 8656483 for much of 2019 (i.e., Hurricane Dorian). Value reported obtained from the NOAA Citizen Weather Observer Program in Beaufort, North Carolina, USA.

During 2010–2020, we surveyed fish communities within seagrass meadows in Back Sound, North Carolina (Fig 1) using a 5-m wide otter trawl (5-m head rope, 2-cm mesh size, 0.6-cm cod end mesh) with no tickler chain [55] on a monthly basis from April-November of each year as part of a long-term ecological study. During each monthly sampling interval, at least six seagrass meadows were trawled. Supplemental trawl sampling was conducted from 2010–2016 as part of separate hypothesis-testing studies [56]. At each meadow, two, 2-min tows were completed, with the entire length of each tow conducted within the meadow. Total travel distance for each tow was recorded based on measurements using a Garmin handheld GPS unit (Garmin International, Olathe, Kansas, USA) and averaged 111 m. All tows were conducted within three hours of a diurnal high tide. After each trawl, all fishes were identified to species, enumerated, and weighed. Because the overwhelming majority of fishes caught in trawls are sizes that would constitute juvenile life stages, we assume that catches are synonymous with nursery role metrics [55]. Surface salinity and water temperature were recorded for each tow using handheld refractometers and thermometers, respectively.

Seagrass surveys were not designed with the current study or long-term fish monitoring survey in mind. Therefore, seagrass surveys were not conducted simultaneously with trawls and do not always match trawl sampling frequencies. However, contemporaneous monthly surveys of seagrass percent cover were performed for related studies at four meadows (designated as SG1-SG4, Fig 1, S1 Table) that were also trawled in May-October in 2016 and from April-September in 2019. In addition, seagrass surveys were conducted at SG2 and SG3 in August and October of 2013 and in May and July of 2014. All surveyed seagrass meadows are mixed species (*Zostera marina*, *Halodule wrightii*, and occasionally *Ruppia maritima*); however, SG1 is dominated by *Z*. *marina*, and SG2-4 is dominated by *H*. *wrightii*. Seagrass surveys were conducted using *in situ* visual estimates of total seagrass percent cover every 2 or 5 m within a quadrat ranging from 0.0625–0.25 m^2^ along a 50-m belt transect located entirely within the meadow. Seagrass surveys conducted in 2016 and 2019 occurred over permanent transects; whereas, surveys in 2013 and 2014 were randomly assigned within the meadow. Seagrass species dominance was calculated as the log ratio of the percent cover of *Z*. *marina* over the percent cover of *H*. *wrightii* + *R*. *maritima*.

Animal and field research was conducted under the North Carolina Division of Marine Fisheries permit # 706481 and University of North Carolina at Chapel Hill Institutional Animal Care and Use Committee protocols # 10–114.0, 13–109.0, and 13–130.0.

### Statistical approach

Given the multitude of ways that fishes and fish community structure may respond to a perturbation as well as confounding factors associated with timing of storm landfall relative to patterns of fish ingress/egress into estuaries, we employed several analytical approaches, data sub-setting, and data transformations (Table 1, S1 Fig). Short-term responses were limited to trawls conducted within a time span of 23 days before and after impact. This window allowed us to compare fish communities observed most proximate to storm fall while (a) preserving balanced sample sizes across treatments and (b) matching the “days between storm event and fish sampling” in control versus impact years. While this temporal window was selected largely out of logistical necessity rather than *a priori* hypotheses *per se* regarding duration of impact, exploring storm impacts within the first ~three weeks post-storm-passage is in line with other studies that have documented short-term responses [28, 57, 58]. Seasonal-scale analyses included all trawls conducted from May-October bisected by storm date, which tended to balance sample sizes between control and impact years given the extended temporal scope and monthly periodicity of trawling.

We presumed that trawls conducted in different years were independent when comparing impact and no impact years, as a new cohort of fishes arrives in the estuary each spring. Because the most abundant fish species, *Lagodon rhomboides* (pinfish), can comprise the majority of trawl catches in North Carolina seagrass meadows (>80%), we calculated two separate metrics of catch per unit effort (CPUE; fish per 100 m towed): one using the entire trawl catch and the other sans *L*. *rhomboides* (hereafter, CPUE and CPUE-Lr, respectively).

### mBACI and BACI

To determine if hurricane passage had an effect on seagrass-associated fish catch rates, species richness, and community structure; we employed a *de facto* multiple before-after-control-impact (mBACI) design following the framework outlined in Hogan et al. (2020), Gericke et al. (2014), Wauchope et al. (2020), and [57, 59, 60]. In this mBACI, we defined “impact” or “storm” years as only those where Saffir Simpson Category 1 or greater windspeeds were observed in the Back Sound study area (Table 2, S1 Fig) during the months of August and September to distinguish hurricanes from ordinary storm events and minimize confounding effects associated with fish ingress/egress. This designated 2011, 2018, 2019, and 2020 as impact/storm years. Years considered as “controls” or “no storm” were defined as those where no tropical storm or cyclone remotely threatened the study area throughout the entire hurricane season (June-November) and included 2013, 2015, and 2017. Years where only tropical storms affected the study area (2010 and 2012) were excluded from mBACI analyses, as environmental impacts of tropical storms are difficult to distinguish from storm events associated with low pressure fronts. Similarly, tropical storms that occurred in the same year as a major hurricane (Hermine in 2016 and Michael in 2018) were not directly examined in the mBACI, as their effects could not be partitioned from the effects of the major hurricane (Matthew in 2016 and Florence in 2018). Within each year, we considered sampling prior to storm events as “before”, and sampling subsequent to storm passage as “after” data. For control years in the mBACI analysis, we applied dummy storm dates of 09/14/2013, 09/06/2015, and 08/08/2017, to designate before- and after periods. These dates were chosen to generally align with observed landfall dates in our dataset (e.g. Florence 09/16/2018, Dorian 09/06/2019, and Isaias 08/04/2020) as well as with peak hurricane season in North Carolina [54].

We conducted two separate BACI analyses to consider the effects of an early- (before August) and a late (after September) storm landing and adjusted our dummy storm dates to match the corresponding hurricane impact date. For these analyses, Hurricane Arthur (7/4/2014) was compared against 2015 (control; dummy storm date: 7/4/2015), and Hurricane Matthew (10/9/2016) was tested against 2017 (control; dummy storm date: 10/9/2017). For both BACIs, we again considered sampling prior to storm arrival as before-impact data, and surveys after storm passage to represent after-impact data.

### ANOVA

We used Analysis of Variance (ANOVA) in R (version 4.0.0) [61] to test for the main and interactive effects of time period (before/after) and year type (control/hurricane) on fish CPUEs and species richness for our mBACI and BACI approaches. This approach is based on the expectation that temporal changes (i.e., short-term or seasonal-scale before-after comparisons, separately) during hurricane years could be statistically different from temporal changes that occur during non-storm years [62]. In this context, the ANOVA term of most interest is the potential interaction between ‘time period’ and ‘year’ [63, 64]. Generating these tests for all possible combinations of hurricane contexts (N = 3; multi-storm mBACI, Arthur BACI, and Matthew BACI), response windows (N = 2; short-term and seasonal), and univariate response metrics (N = 3; CPUE, CPUE-Lr, richness) would result in 18 distinct analyses of means (S1 Fig). We note, however, that we could not run short-term comparisons of means for Hurricane Matthew as the closest trawls conducted prior to Hurricane Matthew were conducted 48 days before storm fall and therefore outside of our short-term temporal window.

While ANOVA is largely robust to violations of normality, data transformations were required in several instances to meet parametric assumptions of homoscedasticity (S1 Fig), which was assessed graphically and accepted when group variances were no larger than 1.5x [65]. Given the large sample size within each factor and group (n = 93 to 239 trawls), parametric tests should be robust to unbalanced sample sizes. As part of our exploratory data analysis, we also examined whether surface water temperatures and salinities taken in concert with trawls differed across BACI treatments.

### Trend analyses

We also conducted time series analyses on seasonal-scale data to explore differences within the mBACI framework and Arthur-specific BACI [60, 66]. Because many time series exhibit patterns through time independently of perturbations, it is possible for fish communities to respond to hurricanes not only by exhibiting a change in mean but with a shift in overall trend. We did not run temporal models for short-term mBACI or for Hurricane Matthew BACI comparisons as surveys were frequently all conducted within one or a few days within the shortened time frames which did not provide enough samples for accurate trend predictions or account for co-occurring effects of seasonality in fish communities.

For each time period-year type treatment and fish metric for the seasonal time frame mBACI, we ran separate generalized additive models (GAMs) against days since storm as the only predicting variable (mgcv library version 1.3–31 [67, 68]). Models were predicted over the temporal range -119 to 80 days since storm for the mBACI and -46 to 115 days since storm for the Arthur-specific BACI and built using a cubic regression spline with penalized shrinkage, a maximum of three degrees of freedom (knots), negative binomial error distribution with log link function, and restricted maximum likelihood (REML) smoothing parameter (y ~ s(Days since Storm), k = 3). For the Arthur-specific trend analysis, we ran negative binomial generalized linear models (GLMs) due to fewer sampling dates during “before” periods. Pairwise differences between control and hurricane-year trends were calculated from predicted model smooths with a 95% confidence interval. Where difference calculations did not overlap zero, we inferred significant difference between control- and hurricane year trends.

### Community structure

We followed the mBACI framework and conducted non-metric multidimensional scaling (NMDS) to examine short-term and seasonal fish communities for Hurricanes Irene, Florence, Dorian, and Isaias combined compared to 2013, 2015, and 2017 combined (Table 2, vegan library version 2.5–6) [69]. We did not compare community structure for pairwise BACI comparisons (i.e., Hurricanes Matthew and Arthur) due to lack of sample size. Community structure analyses were based on a Bray-Curtis extended distance similarity matrix of fish species abundance per trawl sample. Prior to distance calculations, we removed rare species (abundance ≤ 1) [70] and trawls that caught only one fish from the dataset before applying a fourth-root transformation to reduce the influence of extremely abundant species and zero-inflation. A similarity percentages analysis (SIMPER) was used to identify which taxa likely contributed the most to dissimilarity across BACI groups (vegan::simper) [69]. Fish community structure was tested for multivariate homogeneity of group dispersions (PERMDISP) before testing for differences among groups using permutational analysis of variance (PERMANOVA). Water temperature, salinity, days since storm, storm intensity, max gusts, max windspeeds, storm surge, antecedent rainfall, antecedent rainfall anomaly, and storm rainfall were tested for correlation with community ordination and plotted when p < 0.10.

### Tropical cyclone intensities and change

We also gauged how tropical cyclone intensity correlated with storm-coincident shifts in catch and species richness. For this analysis, we included most tropical cyclones that impacted the study area as tropical storms or above on the Saffir Simpson wind scale and examined proportional change in catch and raw change in richness at both short-term (n = 9 storms) and seasonal scales (n = 8 storms). Hurricane Hermine (2016) was excluded from short-term change calculations as no trawl surveys were conducted within a month after impact. Tropical storms Hermine and Michael (2016 and 2018, respectively) were not directly examined in seasonal change comparisons as both (a) reached North Carolina over central portions of the state while transitioning from tropical storms into extratropical cyclones, and (b) occurred in years with other more intense local storms that were used to bisect before and after periods (Table 2).

To isolate the effects of the storm on our study area, we calculated Accumulated Cyclone Energy (ACE) using wind speeds and the duration of time in which the cyclone directly impacted North Carolina. We used ACE as our intensity metric as it more accurately captures the effect and relative difference of a slow- versus fast-moving storm [71, 72]. For this analysis, we calculated proportional change in catch (CPUE and CPUE-Lr) and ran tobit regression models censored at -100% decline against ACE (Table 2, VGAM version 1.1–5, VGAM::vglm). We ran linear regressions of raw change in richness (before-storm minus after-storm data) versus ACE, as richness values were low and averages only ranged between six to eight species.

### Seagrass temporal patterns

Surveys of seagrass percent cover were not conducted at regular intervals; therefore, they do not fit the BACI framework criteria [73]. Instead, mean seagrass percent cover as a function of time is presented with descriptive rather than inferential statistics to provide available context regarding habitat condition before-and-after storms, as well as potential differences in cover between storm versus non-storm years. For descriptive seagrass statistics, we calculated mean percent cover per meadow for each month and year of observation. Similar to previous fish BACIs, we coded control years as those where no storm threatened the study area (2013) and impact years as those where Saffir Simpson Category 1 winds were observed as hurricane years (Arthur 2014, Matthew 2016, and Dorian 2019) bisected by storm date/dummy storm date (Table 2). Only hurricane-year observations were available for meadows SG1 and SG4 (S1 Table).

## Results

### Short-term responses in fish metrics in mBACI design

**Fig 2:**
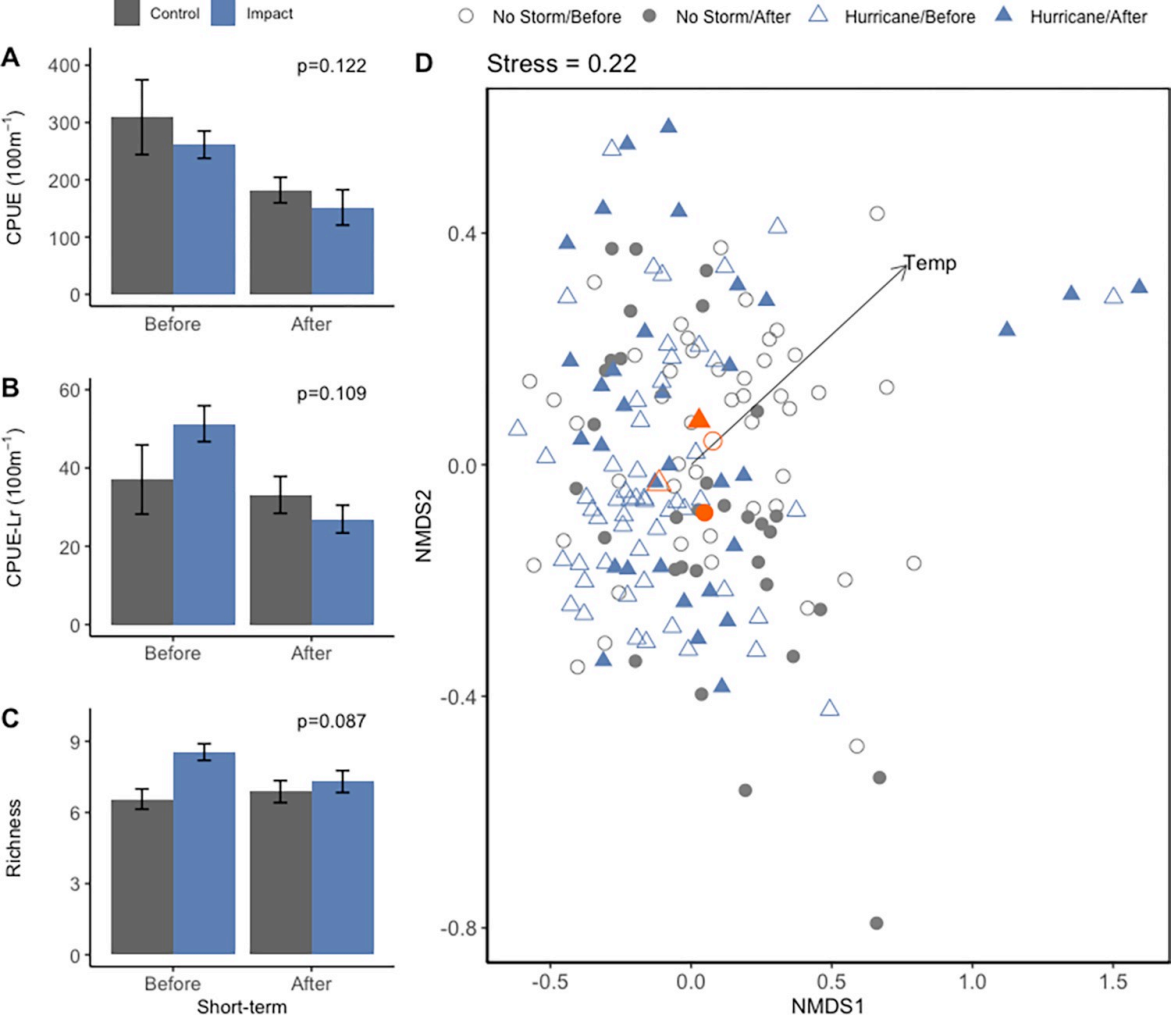
mBACI of short-term fish catches, species richness, and community structure across time periods and year type. Only means are presented for short-term comparisons of catch per unit effort (A); catch per unit effort calculated sans *L*. *rhomboides* (B), and species richness (C). P-values indicate the significance of the ANOVA interaction term. Error bars represent standard error. Non-metric multidimensional scaling (D) of short-term seagrass-associated fish communities based on fourth-root Bray-Curtis extended distances of abundance. Gray coloration and circles indicate control years; blue coloration and triangles indicate impact years. Open symbols indicate before periods and filled symbols indicate after periods. Environmental correlates (p<0.1) are plotted as vectors in the direction of ordination influence. Orange symbols indicate group centroids.

We found no interactive effect of time period and year type on CPUE, CPUE-Lr, or species richness in our mBACI comparisons (ANOVA p_period*year type_ = 0.122 & p_period*year type_ = 0.109, p_period*year type_ = 0.087, respectively, Fig 2A–2C, S2 Table), suggesting that on average, fish catches and richness were not altered by hurricanes. Mean change in CPUE and CPUE-Lr between periods were nearly identical in control and hurricane years (-127 ± 69 & -111 ± 37 CPUE and -4 ± 10 & -22 ± 5 CPUE-Lr, respectively, S3 Table). However, this shift in CPUE-Lr represented a much larger proportional decline in hurricane years (-11% control, -43% impact). Mean richness was higher in hurricane years regardless of period (ANOVA p_year type_ = 0.001, 8.5 species before and 7.5 species after) compared to control years that demonstrated a slight increase in mean species between periods (6.6 species before and 6.9 species after). Water temperatures and salinities taken in concert with trawls did not differ across mBACI groups (ANOVA p_period*year type_ = 0.201, p_period*year type_ = 0.792, respectively, S2 Table).

Fish communities observed within the short-term timeframe differed across time periods and year types (PERMANOVA P_period*year_ = 0.002); and the shift in community structure across year type and time periods was not attributable to differences in within group dispersion (PERMDISP F_3, 160_ = 1.0605, P = 0.3676). However, we found a strong degree of overlap in community ordination across groups using non-metric multi-dimensional scaling (Fig 2D, Stress = 0.22, S2 Fig). Water temperature was the only variable significantly related to ordination and was more strongly correlated with NMDS2 than NMDS1 (p = 0.006, NMDS R^2^ = 0.208, S5 Table). Changes in *L*. *rhomboides*, *Orthopristis chrysoptera* (pigfish), Gerreidae spp. (mojarra species) abundances consistently contributed the most to community dissimilarities, but all four taxa were common in trawls across years and before-or-after storm contexts (S6 Table).

### Seasonal responses in fish metrics in mBACI design

**Fig 3:**
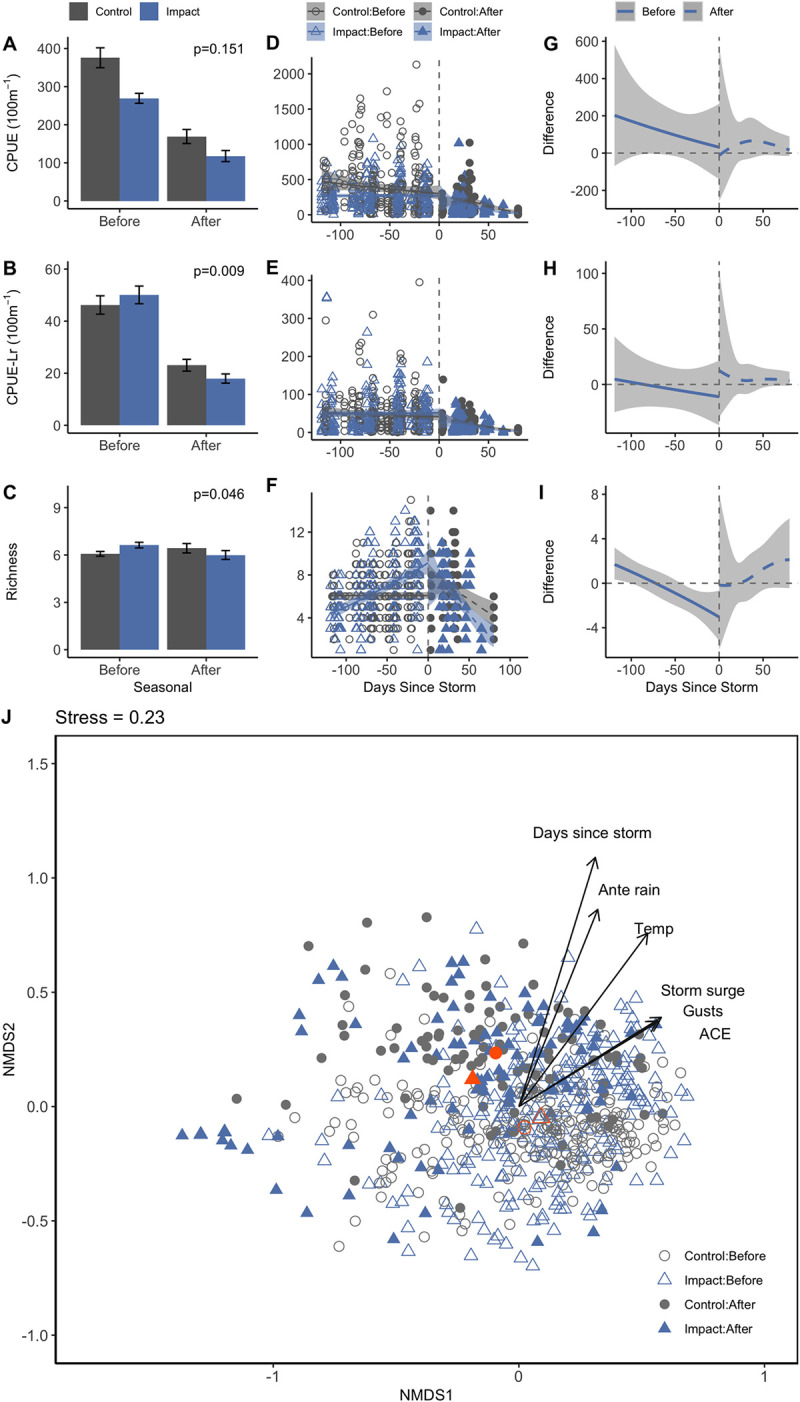
mBACI of seasonal fish catches, species richness, and community structure across time periods and year type. Means (A-C), trend (D-F), and difference between control and impact trends (G-I) are depicted for seasonal comparisons. Confidence intervals and error bars represent 95% confidence and standard errors, respectively. Smoothed lines represent generalized additive models across the seasonal time frame (y ~ s(Days to Storm), k = 3) for both hurricane and storm-free years based on a cubic regression spline with shrinkage. Non-metric multidimensional scaling of seasonal seagrass-associated fish communities (J) are based on fourth-root Bray-Curtis extended distances of abundance. Gray coloration and circles indicate control years; blue coloration and triangles indicate impact years. Open symbols indicate before periods and filled symbols indicate after periods. Environmental correlates (p<0.1) are plotted as vectors in the direction of ordination influence. Orange symbols indicate group centroids.

We found that CPUE was not statistically different across time period and year type, but CPUE-Lr and species richness were (CPUE p_period*year_ = 0.151, CPUE-Lr p_period*year_ = 0.009, Richness p_period*year_ = 0.046, Fig 3A and 3B, S7 Table). Despite the statistical significance of these metrics, mean changes between periods suggest small to negligible shifts in raw fish counts and richness. On average, seasonal CPUE and CPUE-Lr declined by comparable amounts between periods in both control and hurricane years (-207 ± 32 and -153 ± 18 CPUE, and -22 ± 4, -33 ± 5 CPUE -Lr, S4 Table). Proportionally, this represented only a 1% greater decline in CPUE and a 14% greater decline in CPUE-Lr during hurricane years than during non-storm years. Mean richness across seasons also did not exhibit a distinct ecological shift and ranged from 6.1 to 6.7 species per tow regardless of time period or year type. At the seasonal timescale, water temperatures and salinities taken in concert with trawls also did not differ across mBACI groups (ANOVA p_period*year type_ = 0.201, p_period*year type_ = 0.792, respectively, S2 Table).

Based on the GAM analysis, catch trends (CPUE and CPUE-Lr) did not differ across control and hurricane years (Fig 3D and 3E). Notably, CPUE, CPUE-Lr, and richness trends in after periods consistently overlapped and exhibited similar model directions and shapes (p ≤ 0.001 for all “after” period models, Fig 3G–3I). The smoothing term, days since storm, did not significantly explain CPUE or CPUE-Lr trends during “before” periods of hurricane years (p = 0.356, p = 0.426, S8 Table) or CPUE-Lr and species richness trends during “before” periods of control years (p = 0.129, p = 0.466). Species richness trends during “before” periods exhibited dissimilar trends across year types with richness trending steady at roughly six species during control years and increasing from roughly four to nine species during hurricane years (Fig 3F and 3I).

Fish communities observed across the seasonal timeframe differed across time periods and year types (PERMANOVA P_period*year_ = 0.001, Fig 3J); and the shift in community structure across year type and time periods was attributable to differences in within group dispersion (PERMDISP F_3, 647_ = 7.7587, P < 0.001). However, there was a large degree of overlap across mBACI groups with communities differing primarily across time period observed and not year type (S3 Fig). Days since storm, and antecedent rainfall significantly influenced community ordination along NMDS2 (p = 0.001 & R^2^ = 0.554, p = 0.001 & R^2^ = 0.351, respectively, S5 Table). Similar to the short-term analyses, *L*. *rhomboides*, *O*. *chrysoptera*, Gerreidae spp., and *Leiostomus xanthurus* (spot), contributed the most to community dissimilarities but all four were common across year types and time periods (S6 Table).

### Early- and late-season cyclones in BACI design

When comparing Hurricane Arthur (July 2014) to an analogous control year (2015), we found no interactive effect of time period or year type on any univariate fish metric across the short-term (CPUE p_period*year_ = 0.210, CPUE-Lr p_period*year_ = 0.271, Richness p_period*year_ = 0.797, S3 Table, S4 Fig) or seasonal scale (CPUE p_period*year_ = 0.614, CPUE-Lr p_period*year_ = 0.068, Richness p_period*year_ = 0.519, S7 Table, S4 Fig). Similarly, we found that CPUE, CPUE-Lr, and species richness trends in 2014 did not differ compared to 2015 (S9 Table, S4 Fig).

When comparing Hurricane Matthew (October 2016) to 2017 as the control year, we found an interactive effect of time period and year type on seasonal CPUE but not CPUE-Lr or species richness (CPUE p_period*year_ = 0.011, CPUE-Lr p_period*year_ = 0.364, Richness p_period*year_ = 0.136, S7 Table, S5 Fig). The statistical significance of CPUE across groups was likely driven by the much larger average catch observed before Hurricane Matthew (277 ± 29 fish 100 m^-1^ S4 Table) compared to the same period in 2017 (94 ± 17 fish 100 m^-1^), as mean catches during after periods were not substantially different between year types (33 ± 9 fish 100 m^-1^ after Matthew and 28 ± 5 fish 100 m^-1^ “after” 2017).

### Changes corresponding with cyclone intensity

**Fig 4:**
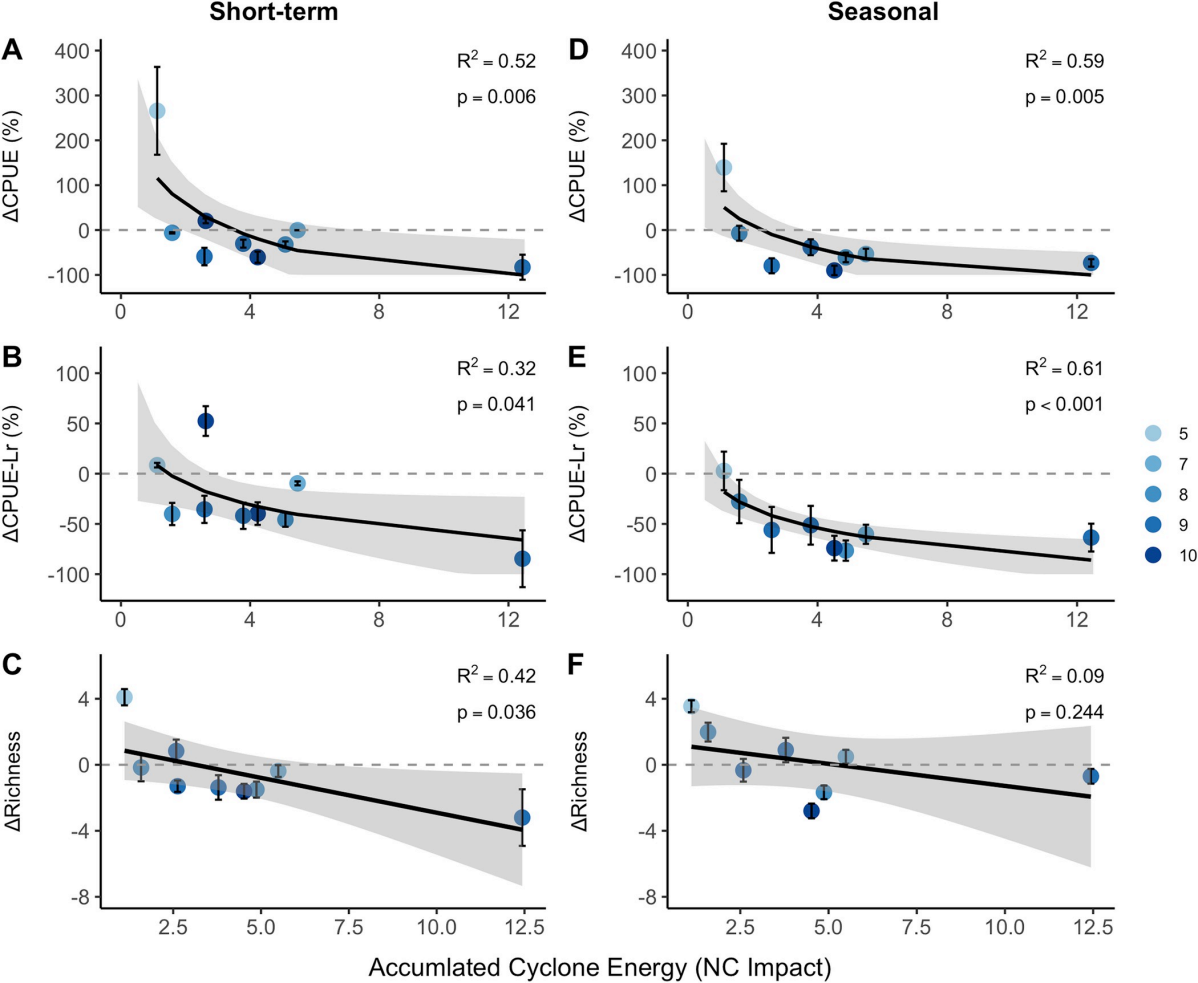
Change in fish metrics as a function of cyclone (tropical storms and hurricanes) intensity. Short-term changes are depicted column 1, and seasonal-scale changes are represented in column 2. A & D) Proportional change in catch per unit effort. B & E) Proportional change in catch per unit effort sans *L*. *rhomboides*, and C & F) change in raw species richness. Models of CPUE and CPUE-Lr represent a tobit regression censored at -100%, and linear regressions are plotted for change in raw species richness. Model shading indicates 95% confidence intervals also censored at -100% for catch metrics. Point coloration (light blue to dark blue) indicates increasing month of impact.

We found that accumulated cyclone energy was positively correlated with the amount of change in CPUE, CPUE-Lr, and species richness at both the short-term and seasonal time frame, with more intense storms associated with greater declines in each of these metrics (Fig 4). This relationship was particularly pronounced for changes within short-term time frame (R^2^_CPUE_ = 0.52 & p = 0.006; R^2^_CPUE-Lr_ = 0.33 & p = 0.041; R^2^_richness_ = 0.42 & p = 0.036, Fig 4A–4C). CPUE and CPUE-Lr consistently decreased after cyclone impact with two notable exceptions. The only storms where CPUE and CPUE-Lr demonstrated increases were 1) after Tropical Storm Beryl, which was the weakest cyclone and occurred in late spring (5/30/2011), coinciding with seasonal fish ingress into the estuary, and; 2) after Tropical Storm Michael (10/11/18), which occurred less than one month after Hurricane Florence on 9/14/2019. Florence was the strongest storm that impacted Back Sound over the study duration (substantially higher in ACE, rainfall, and storm surge than all other cyclones in our study) and was associated with the greatest short-term decline in CPUE, CPUE-Lr, and species richness, but remained comparable in effect to storms of much lower intensity at the seasonal time frame.

### Seagrass trends

**Fig 5:**
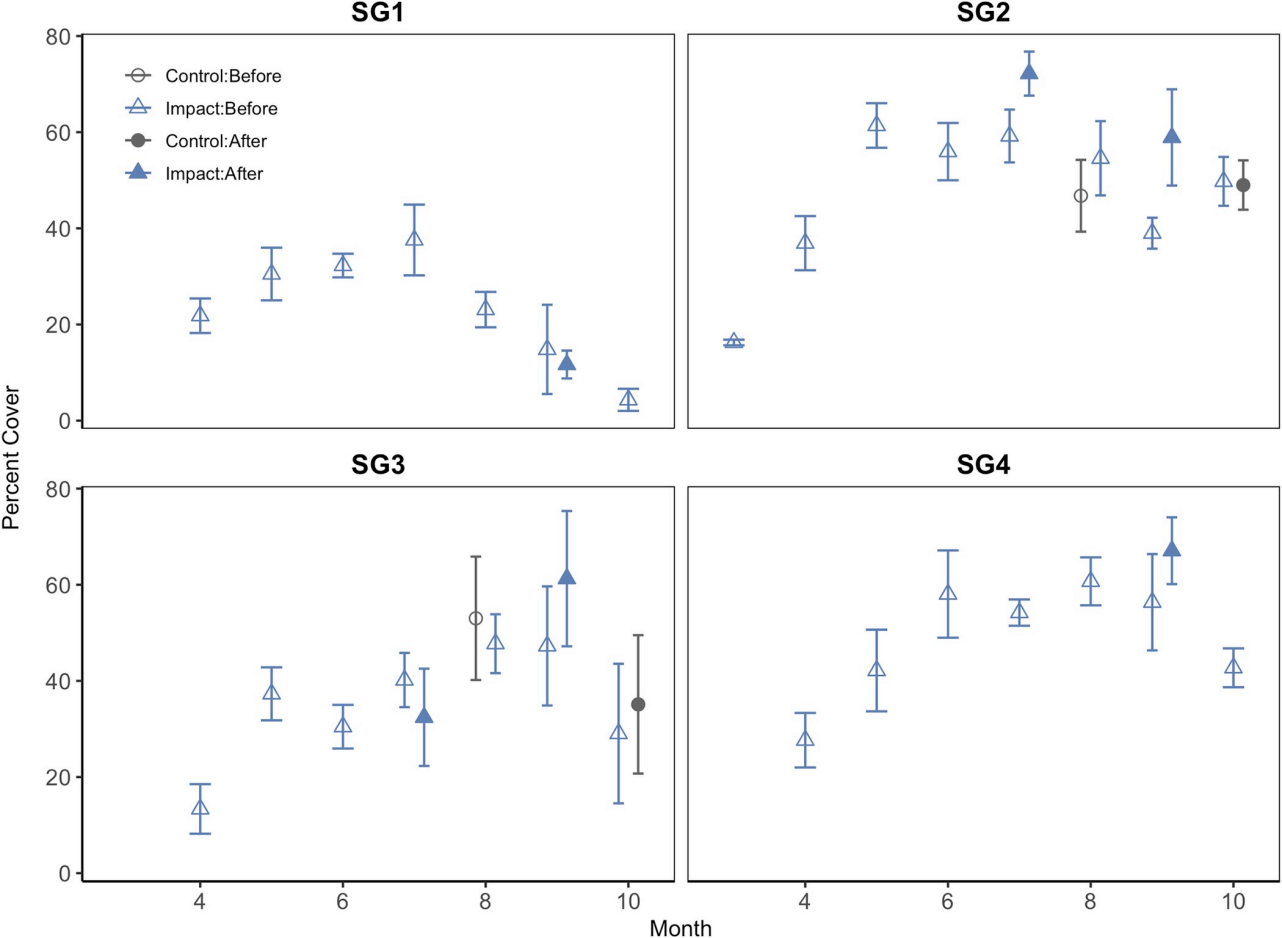
Trends in seagrass percent cover by month and BACI treatment for individual meadows. Gray coloration and circles indicate control year treatments; blue coloration and triangles indicate hurricane years. Open symbols indicate measurements taken before hurricanes, and filled symbols indicate measurements taken after storms.

Seagrass percent cover displayed consistent seasonal trends associated with productivity patterns of the dominant species within the meadow regardless of year type (Fig 5, S1 Table). Overall, percent cover at surveyed meadows appeared to be stable from 2014–2019. Given the non-standardized sampling frequency, percent cover at SG1 (a meadow that is *Z*. *marina-*dominated) after hurricane impact in September 2019 did not differ noticeably from measurements obtained in September 2016 before Hurricane Matthew (before impact). At sites where *H*. *wrightii* is the dominant seagrass species (SG2-SG4), percent cover of seagrass during the month of September was often greater after impact (September 2019, after Hurricane Dorian) than that observed prior to impact (September 2016, before Hurricane Matthew). This divergence was greatest at site SG2 where cover averaged 39 ± 3% in September 2016 and 59 ± 10% in September 2019. We also did not detect a consistent effect of hurricanes on seagrass cover during the month of July. Percent cover after Hurricane Arthur in July 2016 was greater at SG2 but slightly less at SG3 compared to average measurements taken in July prior to storms in 2016 and 2019.

## Discussion

Coastal ecosystems have evolved and adapted to withstand a wide array of environmental conditions and periodic disturbances due to natural climate variability [74]. However, given the likelihood of increasing storm intensity as a result of climate change [75, 76], it is pivotal to consider how these acute pulse disturbances, from which ecosystems can recover, may compound to function more similarly to chronic, press disturbances that push the system towards an alternative state [1, 2]. Hurricanes are predicted to increase in both rainfall and windspeed and potentially impact larger geographical swaths, leading to more devastating impacts across socio-ecological systems [77]. Our study evaluates how seagrass-associated fish communities within a temperate-subtropical estuary along the western Atlantic Ocean have responded after a 10-year period with 10 tropical cyclones. Overall, our findings indicate that fishes were generally resistant to storm impacts across multiple tropical cyclones, with no substantial differences in short-term or seasonal patterns of catch or community structure during storm versus no-storm years. However, increasing storm intensity was correlated with declines in fish catches at the short-term and seasonal scale with greater proportional changes in catches calculated sans *L*. *rhomboides*. The overall stability of the fish community was likely underpinned by seagrass meadow persistence, indicating an ability to provide nursery habitat despite multiple notable disturbances. However, if hurricanes increase in intensity, duration, or frequency, it may lead to long-term, large-scale ecosystem shifts that are indicative of press rather than pulse disturbances.

The physical impact of hurricanes can be patchy across space and cause extensive damage in some areas and leave others unaltered [36]. Estuarine seagrass meadows may be more susceptible to negative indirect effects of stormwater runoff and retention, whereas meadows without offshore barrier islands or coral reefs are more likely to be impacted by physical wave scour [78]. Indeed, studies have documented variable impacts on seagrass meadows after tropical cyclones and severe storms including no effect, change in species composition, faster growth, and both long and short-term declines in area [37, 42, 45, 79, 80]. Provided the disturbance does not cause severe habitat degradation, fishes may rapidly repopulate areas [58, 81–83]. We did not detect an obvious decline in seagrass percent cover at four meadows in our system, which may have buffered against drastic shifts in fish community catches and structure. However, we note that this is a limited representation of general seagrass trends across the estuary and long-term surveys are needed to assess habitat trends over time [84]. Seagrass meadows in the temperate-subtropical estuaries of North Carolina are composed of a mix of “weedy” or fast-growing species (i.e. *H*. *wrightii* and *R*. *maritima*) that can quickly recolonize after disturbance as well as *Z*. *marina* that utilizes a mixed-annual reproductive strategy that may enhance seagrass resistance and recovery from physical stressors [85–87]. Given that our trawls were conducted entirely within the seagrass meadow, our surveys may be less sensitive to changes in overall seagrass area. Our results demonstrate that the unit-area function of seagrass persisted [32], but estimates of the overall change in meadow area from 2010–2020 would provide a clearer indication of regional ecosystem resistance and recovery to pulse stressors [88].

In general, estuarine communities are capable of withstanding shifts in salinity [89–91]; however, resilience and ability to adjust to rapidly decreasing or sustained anomalous salinity varies by species [92, 93] and magnitude of perturbation. Antecedent meteorological conditions, such as drought, excessive rainfall, and/or sustained high water temperatures prior to cyclones can affect habitat quality and nursery function prior to hurricane impact. We found that seasonal community structure was most strongly affected by temperature, days since storm, antecedent rainfall, and storm intensity characteristics. However, all environmental covariates were only weakly correlated with community structure, and communities were largely similar irrespective of storm impact. Indeed, previous studies have documented post-hurricane shifts in estuarine nekton species towards both more oligohaline species [22] as well as brackish species [21]. Water temperatures and salinities taken in concert with trawls were not different across impact or time period in our study (S2 Table); however, all trawls conducted post-hurricane were 5 or more days after passage, at which time salinity signals may have dissipated due to our study site proximity to inlets and oceanic influences.

Our findings generally align with the majority of studies (Table 1) that found no distinct effect of hurricane(s) on fish community structure. Fishes that occupy inshore seagrass meadows as juveniles and spend their adult life stages offshore may be buffered from cyclones due to the coinciding timing of migrations to deeper waters offshore with peak hurricane season in North Carolina [27]. We did not find a strong effect of seasonal timing on the overall impact of the storm on fish catch and community structure that was markedly different from control years. However, seasonality may have a different effect in tropical regions where patterns of fish abundance do not fluctuate as substantially. Catches increased in the one-month period between Hurricane Florence (Sept 14) and Tropical Storm Michael, which indicates that despite the effects of Tropical Storm Michael on top of the notable negative impact of Hurricane Florence, fishes were actively repopulating seagrass meadows during peak Atlantic hurricane season. Studies have suggested that storms and other large disturbances may alleviate fishing pressure and potentially promote recovery [94, 95], and in nutrient-poor ecosystems, cyclone-induced upwelling and river discharge can also lead to increases in fish catches [96].

Regardless of disturbance events, there is extensive natural variability in fish population dynamics [97], as evidenced in our study by the wide range of catch in both hurricane and control years during “before” periods. Large-scale climactic processes that influence hurricane activity, local meteorology, and biophysical conditions, such as the Atlantic Multidecadal Oscillation (AMO), can also influence fish populations separately from the direct impact of the storm itself. El Niño Southern Oscillation (ENSO) can heavily influence fisheries in the Pacific Ocean [98], and the AMO can modulate fish abundances in the eastern Atlantic [99]. However, few linkages have been investigated or demonstrated between the AMO and fish populations in estuarine or coastal waters of the western Atlantic [100], and rigorous long-term surveys are needed to examine the interactive effects of climactic oscillations on disturbances and marine ecosystem structure. Our study presents a case study of coastal ecosystem response to cyclones at a unique biogeographic transition zone that may be particularly sensitive to climate-induced shifts in community structure [87, 101] and may serve as a framework for future examinations of tropical cyclones under a global change scenario.

While our data provide reason for optimism regarding seagrass and seagrass-associated fish persistence after multiple cyclones, several climate forecasts and models have indicated that hurricane intensity and associated rainfall will increase in response to rising sea surface temperatures [75, 76, 102, 103], and greater cyclone magnitude was found to correlate strongly with greater declines in fish catch. If return intervals between major tropical cyclones become shorter than those needed for community recovery, the concept of individual storms as acute, pulse disturbances may need to be reframed. Our study and many others examine cyclones as isolated pulse disturbances, but their effects are compounded by and intertwined with escalating press stressors associated with climate change [104], habitat degradation [105], and fishing pressure [106]. Combined, these stressors may lead to an erosion of estuarine ecosystem resistance and resilience on timelines more similar to those of ramp disturbances that increase in intensity over time and/or press disturbances from which the ecosystem may not recover [7]. Studies that provide a single snapshot of before and after a single event can lead to misleading findings if multi-year trends are ignored [107]. Long-term syntheses geared towards understanding how global change will impact disturbance characteristics and collectively push ecosystems towards alternative states are necessary for effective coastal management, especially as society increasingly moves towards building resilient ecosystems [108].

## Supporting information

S1 Appendix Literature review citations for Table 1. Published studies that examined change in fish abundances before and after hurricanes listed in the literature review.
(PDF)

S1 Table Seagrass meadow locations and survey dates. Meadows surveyed for both fishes and percent cover of seagrass. Seagrass survey dates (month/year) and periodicity listed.
(PDF)

S2 Table Temperature and salinity ANOVA summary statistics. Summary results examining potential correlations between environmental variables, namely surface water temperature and salinity, fish catches and species richness.
(PDF)

S3 Table Short-term ANOVA summary statistics. ANOVA results for short-term time frame analyses conducted for multi-storm mBACI and Arthur BACI. Post-hoc test results are not presented as no interaction term was significant.
(PDF)

S4 TableMean fish metrics across mBACI treatments.Mean +/- standard error CPUE, CPUE-Lr, and species richness for multi-storm mBACI and BACI comparisons. Percent change is calculated as the decline or increase in catch or richness between before and after periods: $\frac{before-after}{before}\times 100$. Note that means presented are rounded to whole values for catch and one decimal place for species richness, and percent change presented was calculated using unrounded values.(PDF)

S5 Table NMDS environmental correlates. Summary results of environmental variables tested for correlation with fish community structure for both short-term and seasonal time frames.
(PDF)

S6 Table Contributing species to group dissimilarities. Results of Similarity Percentages (SIMPER) analysis indicating the species that contribute the most to dissimilarity across BACI groups based on Bray-Curtis dissimilarities calculated from fourth-root transformed abundance data. Only the top 10 species that contribute the most to between-group dissimilarities are listed.
(PDF)

S7 Table Seasonal ANOVA and Tukey HSD summary statistics. ANOVA results for seasonal time frame analyses conducted for multi-storm mBACI, Arthur BACI, and Matthew BACI. Post-hoc test results are presented when the interaction term is significant at p<0.05.
(PDF)

S8 Table GAM summary statistics for seasonal mBACI. All generalized additive models (GAMs) were run for the seasonal time frame against days since storm as the independent variable and built using a cubic regression spline with penalized shrinkage, a maximum of three degrees of freedoms, negative binomial error distribution with log link function, and restricted maximum likelihood smoothing parameter. edf = effective degrees of freedom, logLik = log likelihood, Dev = deviance, df.r = residual degrees of freedom, AIC = Akaike information criterion BIC = Bayesian information criterion.
(PDF)

S9 Table Hurricane Arthur GLM summary statistics. Negative binomial generalized linear models for seasonal-scale trend analysis of Hurricane Arthur (2014) versus 2015 as the control year. Est = estimate, df.r = residual degrees of freedom, AIC = Akaike information criterion BIC = Bayesian information criterion.
(PDF)

S1 Fig Statistical analysis flowchart and transformations. Decision-tree indicating how fish data was subsetted, transformed, and analyzed. If not listed as transformed, raw data was used as the response variable.
(PDF)

S2 Fig Individual mBACI treatment PCoA ordinations of short-term communities. This figure demonstrates the potential difference/lack of difference in short-term community dispersion within each mBACI treatment using Principle Coordinates Analysis. Convex hulls are drawn in dashed lines through the outer-must points.
(PDF)

S3 Fig Individual mBACI group PCoA ordinations of seasonal communities. This figure demonstrates the potential difference/lack of difference in seasonal community dispersion within each mBACI treatment using Principle Coordinates Analysis. Convex hulls are drawn in dashed lines through the outer-must points.
(PDF)

S4 Fig Hurricane Arthur BACI comparisons. Short-term and seasonal fish catches and species richness across time periods (before vs. after) and year type (control vs. impact) for Hurricane Arthur (July 2014) compared to 2015 (control year). Only means are presented for short-term comparisons (column 1); whereas means, trend, and difference between control and impact trends (columns 2–4, respectively) are depicted for seasonal comparisons. Catch per unit effort (CPUE) is presented in row 1 (A, D, H, K); CPUE calculated sans *L*. *rhomboides* is presented in row 2 (B, E. I, L), and species richness is row 3 (C, F, J, M). P-values indicate the significance of the interactive ANOVA term. Error bars represent standard error. Smoothed lines represent negative binomial generalized linear models for both hurricane and storm-free years with 95% confidence intervals.
(PDF)

S5 Fig Hurricane Matthew seasonal BACI comparisons. Seasonal fish catch per unit effort (A), catch per unit effort sans *Lagodon rhomboides* (B) and species richness (C) across time periods (before vs. after) and year type (control vs. impact) for Hurricane Matthew (October 2016) compared to 2017 (control year). Only means are presented for seasonal comparisons (column 1), as the closest trawl samples prior to hurricane Matthew occurred outside of the short-term window and all trawls that occurred after the storm were conducted on the same day. P-values indicate the significance of the interactive ANOVA term. Error bars represent standard error.
(PDF)
