# Supplementary material for: Tropical cyclone impacts on seagrass-associated fishes in a temperate-subtropical estuary
Source: PLoS One. 2022 Oct 13;17(10):e0273556. doi: 10.1371/journal.pone.0273556 (PMC9560482; doi:10.1371/journal.pone.0273556)
Supplement: S1 Table — Meadows surveyed for both fishes and percent cover of seagrass. Seagrass survey dates (month/year) and periodicity listed. (PDF) [file pone.0273556.s002.pdf]

# Supplementary Information

**S1 Appendix. Literature Review Citations for Table 1**
Published studies that examined change in fish abundances before and after hurricanes listed in the literature review.

**S1 Figure. Statistical Analysis Flowchart and Transformations**
Decision-tree indicating how fish data was subsetted, transformed, and analyzed. If not listed as transformed, raw data was used as the response variable.

**S1 Table. Seagrass meadow locations and survey dates**
Meadows surveyed for both fishes and percent cover of seagrass. Seagrass survey dates (month/year) and periodicity listed.

**S2 Table. Temperature and Salinity ANOVA summary statistics**
Summary results examining potential correlations between environmental variables, namely surface water temperature and salinity, fish catches and species richness.

**S3 Table. Short-term ANOVA mBACI summary statistics**
Summary results of short-term ANOVA tests across time period (before vs. after) and year type (control vs. impact year) for mBACI and Arthur BACI. Short-term analyses include only trawls conducted within 23 days of stormfall. Response variables were transformed when necessary to meet assumptions of parametric statistics. Post-hoc test results are not presented as no interaction term was significant.

**S4 Table. Mean fish metrics across mBACI treatments**
Mean +/- standard error CPUE, CPUE-Lr, and species richness for multi-storm mBACI and BACI comparisons. Percent change is calculated as the decline or increase in catch or richness between before and after periods: $\frac{\text{before} - \text{after}}{\text{before}} \times 100$. **Note:** Means presented are rounded to whole values for catch and one decimal place for species richness. Percent change was calculated using unrounded values and may differ from calculations based upon means in the table.

**S5 Table. NMDS environmental correlates**
Summary results of environmental variables tested for potential correlation with fish community structure at the short-term and seasonal time frames.

**S6 Table. Contributing species to group dissimilarities**
Results of Similarity Percentages (SIMPER) analysis indicating the species that contribute the most to dissimilarity across BACI groups based on Bray-Curtis dissimilarities calculated from fourth-root transformed abundance data. Only the top 10 species that contribute the most to between-group dissimilarities are listed.

**S7 Table. Seasonal ANOVA and Tukey HSD for mBACI, Arthur- and Matthew BACIs**
Summary results of seasonal-scale ANOVA tests across time period (before vs. after) and year type (control vs. impact year). Seasonal analyses include trawls conducted during the months of

May-October. Response variables were transformed when necessary to meet assumptions of parametric statistics. Post-hoc test results are presented when the interaction term is significant at $p<0.05$. Abbreviations indicate treatments; CB = control before, IB = impact before, CA = control after, IA = impact after

**S8 Table. GAM Model Summary Statistics for Seasonal mBACI and Hurricane Arthur**
All GAM models were run for the seasonal time frame against days since storm as the independent variable and built using a cubic regression spline with penalized shrinkage, a maximum of three degrees of freedoms, negative binomial error distribution with log link function, and restricted maximum likelihood smoothing parameter. edf = effective degrees of freedom, logLik = log likelihood, Dev = deviance, df.r = residual degrees of freedom, AIC = Akaike information criterion BIC = Bayesian information criterion.

**S2 Figure. Individual mBACI treatment PCoA ordinations of short-term communities**
This figure demonstrates the potential difference/lack of difference in short-term community dispersion within each mBACI treatment using Principle Coordinates Analysis. Convex hulls are drawn in dashed lines through the outer-must points.

**S3 Figure. Individual mBACI group PCoA ordinations of seasonal communities**
This figure demonstrates the potential difference/lack of difference in seasonal community dispersion within each mBACI treatment using Principle Coordinates Analysis. Convex hulls are drawn in dashed lines through the outer-must points.

**S4 Figure. Hurricane Arthur BACI comparisons**
Short-term and seasonal fish catches and species richness across time periods (before vs. after) and year type (control vs. impact) for Hurricane Arthur (July 2014) compared to 2015 (control year). Only means are presented for short-term comparisons (column 1); whereas means, trend, and difference between control and impact trends (columns 2-4, respectively) are depicted for seasonal comparisons. Catch per unit effort (CPUE) is presented in row 1 (A, D, H, K); CPUE calculated sans *L. rhomboides* is presented in row 2 (B, E. I, L), and species richness is row 3 (C, F, J, M). P-values indicate the significance of the interactive ANOVA term. Error bars represent standard error. Smoothed lines represent generalized additive models ($y \sim s(\text{Days to Storm})$, $k = 3$) for both hurricane and storm-free years based on a cubic regression spline with shrinkage and 95% confidence intervals.

**S5 Figure. Hurricane Matthew Seasonal BACI comparisons**
Seasonal fish catch per unit effort (A), catch per unit effort sans *Lagodon rhomboides* (B) and species richness (C) across time periods (before vs. after) and year type (control vs. impact) for Hurricane Matthew (October 2016) compared to 2017 (control year). Only means are presented for seasonal comparisons (column 1), as the closest trawl samples prior to hurricane Matthew occurred outside of the short-term window and all trawls that occurred after the storm were conducted on the same day. P-values indicate the significance of the interactive ANOVA term. Error bars represent standard error.

**S1 Appendix. Literature Review Citations for Table 1**
Published studies that examined change in fish abundances before and after hurricanes listed in the literature review.

1. Adams A. Effects of a hurricane on two assemblages of coral reef fishes: Multiple-year analysis reverses a false "snapshot" interpretation. Bull Mar Sci. 2001;69: 341–356.
2. Adams AJ, Ebersole JP. Resistance of coral reef fishes in back reef and lagoon habitats to a hurricane. Bull Mar Sci. 2004;75: 101–113.
3. Anton A, Cebrian J, Duarte CM, Heck Jr KL, Goff J. Low impact of Hurricane Katrina on seagrass community structure and functioning in the northern Gulf of Mexico. Bull Mar Sci. 2009;85: 45–59.
4. Bortone SA. Effects of a hurricane on the fish fauna at Destin, Florida. Flor Sci. 1976; 245–248.
5. Bouchon C, Bouchon-Navaro Y, Louis M. Changes in the coastal fish communities following hurricane Hugo in Guadelope Island (French West Indies). Atoll Res Bull. 1994.
6. Burkholder J, Eggleston D, Glasgow H, Brownie C, Reed R, Janowitz G, et al. Comparative impacts of two major hurricane seasons on the Neuse River and western Pamlico Sound ecosystems. Proc Natl Acad Sci USA. 2004;101: 9291–9296.
7. Cheal A, Coleman G, Delean S, Miller I, Osborne K, Sweatman H. Responses of coral and fish assemblages to a severe but short-lived tropical cyclone on the Great Barrier Reef, Australia. Coral Reefs. 2002;21: 131–142.
8. Davis J, Laird B. The effects of tropical storm Agnes on the Chesapeake Bay estuarine system. The Chesapeake Reserach Consortium; 1976. Report No.: CRC Publication No. 54.
9. Dolloff CA, Flebbe PA, Owen MD. Fish habitat and fish populations in a southern Appalachian watershed before and after Hurricane Hugo. Trans Am Fish Soc. 1994;123: 668–678.
10. Fenner DP. Effects of Hurricane Gilbert on Coral Reefs, Fishes and Sponges at Cozumel, Mexico. Bull Mar Sci. 1991;48: 719–730.
11. Fitzsimons JM, Nishimoto RT. Use of fish behavior in assessing the effects of Hurricane Iniki on the Hawaiian island of Kaua'i. Environ Biol Fishes. 1995;43: 39–50.
12. Greenwood MF, Stevens PW, Matheson RE. Effects of the 2004 hurricanes on the fish assemblages in two proximate southwest Florida estuaries: change in the context of interannual variability. Estuaries Coast. 2006;29: 985–996.
13. Lassig BR. The effects of a cyclonic storm on coral reef fish assemblages. Environ Biol Fishes. 1983;9: 55–63.
14. Letourneur Y, Harmelin-Vivien M, Galzin R. Impact of hurricane Firinga on fish community structure on fringing reefs of Reunion Island, S.W. Indian Ocean. Environ Biol Fishes. 1993;37: 109–120. doi:10.1007/BF00000586
15. Locascio JV, Mann DA. Effects of Hurricane Charley on fish chorusing. Biol Lett. 2005;1: 362–365.

16. Paerl HW, Bales JD, Ausley LW, Buzzelli CP, Crowder LB, Eby LA, et al. Ecosystem impacts of three sequential hurricanes (Dennis, Floyd, and Irene) on the United States' largest lagoonal estuary, Pamlico Sound, NC. Proc Natl Acad Sci USA. 2001;98: 5655–5660.

17. Paperno R, Tremain D, Adams D, Sebastian A, Sauer J, Dutka-Gianelli J. The disruption and recovery of fish communities in the Indian River Lagoon, Florida, following two hurricanes in 2004. Estuaries Coast. 2006;29: 1004–1010.

18. Springer VG, McErlean AJ. A study of the behavior of some tagged south Florida coral reef fishes. Am Midl Nat. 1962; 386–397.

19. Stevens PW, Blewett DA, Casey JP. Short-term effects of a low dissolved oxygen event on estuarine fish assemblages following the passage of Hurricane Charley. Estuaries Coast. 2006;29: 997–1003.

20. Switzer T, Winner B, Dunham N, Whittington J, Thomas M. Influence of sequential hurricanes on nekton communities in a southeast Florida estuary: short-term effects in the context of historical variations in freshwater inflow. Estuaries Coast. 2006;29: 1011–1018.

21. Yu J, Tang D, Chen G, Li Y, Huang Z, Wang S. The positive effects of typhoons on the fish CPUE in the South China Sea. Cont Shelf Res. 2014;84: 1–12. doi:10.1016/j.csr.2014.04.025

22. Yu J, Tang D, Li Y, Huang Z, Chen G. Increase in fish abundance during two typhoons in the South China Sea. Adv Space Res. 2013;51: 1734–1749. doi:10.1016/j.asr.2012.11.019

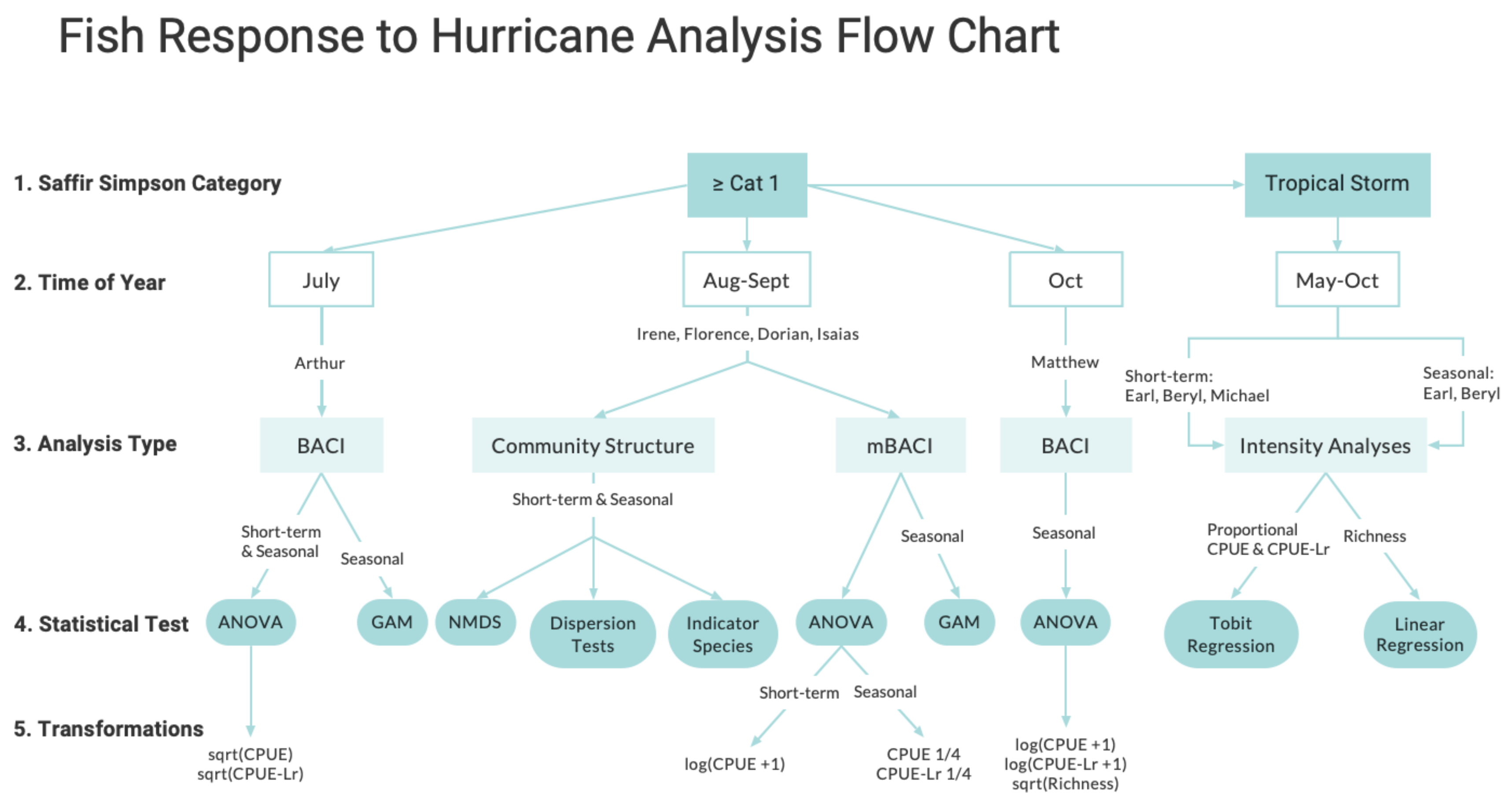


**S1 Figure. Statistical Analysis Flowchart and Transformations**
Decision-tree indicating how fish data was subsetted, transformed, and analyzed. If not listed as transformed, raw data was used as the response variable.

**S1 Table. Seagrass meadow locations and survey dates**

| **Meadow** | **Latitude** | **Longitude** | **Dominant Seagrass spp.** | **Surveys** |
|---|---|---|---|---|
| SG 1 | 34.691747 | -76.622573 | *Zostera marina* | May-Oct 2016 (monthly)<br>Apr-Sep 2019 (monthly) |
| SG 2 | 34.697874 | -76.595503 | *Halodule wrightii* | Aug & Oct 2013<br>May & July 2014<br>May-Oct 2016 (monthly)<br>Apr-Sep 2019 (monthly) |
| SG 3 | 34.699899 | -76.592917 | *Halodule wrightii* | Aug & Oct 2013<br>May & July 2014<br>May-Oct 2016 (monthly)<br>Apr-Sep 2019 (monthly) |
| SG 4 | 34.703403 | -76.587869 | *Halodule wrightii* | May-Oct 2016 (monthly)<br>Apr-Sep 2019 (monthly) |

Meadows surveyed for both fishes and percent cover of seagrass. Seagrass survey dates (month/year) and periodicity listed.

**S2 Table. Temperature and Salinity ANOVA summary statistics**

| | Time Frame | Factor | Df | Sum Sq | Mean Sq | F value | P |
|---|---|---|---|---|---|---|---|
| **Temperature** | **Short-term** | Period | 1 | 26.3 | 26.28 | 1.741 | 0.189 |
| | | Year Type | 1 | 18 | 19.98 | 1.191 | 0.277 |
| | | Period*Year Type | 1 | 24.9 | 24.94 | 1.652 | 0.201 |
| | | Residuals | 150 | 2264.6 | 15.1 | | |
| | **Seasonal** | Period | 1 | 54 | 53.83 | 3.775 | 0.052 |
| | | Year Type | 1 | 45 | 45.31 | 3.178 | 0.075 |
| | | Period*Year Type | 1 | 25 | 24.51 | 1.719 | 0.190 |
| | | Residuals | 603 | 8597 | 14.26 | | |
| **Salinity** | **Short-term** | Period | 1 | 2.3 | 2.267 | 0.493 | 0.484 |
| | | Year Type | 1 | 6.2 | 6.16 | 1.341 | 0.249 |
| | | Period*Year Type | 1 | 0.3 | 0.321 | 0.1 | 0.792 |
| | | Residuals | 142 | 652.4 | 4.594 | | |
| | **Seasonal** | Period | 1 | 0 | 0.196 | 0.031 | 0.859 |
| | | Year Type | 1 | 7 | 7.191 | 1.154 | 0.283 |
| | | Period*Year Type | 1 | 22 | 22.286 | 3.575 | 0.060 |
| | | Residuals | 549 | 3422 | 6.234 | | |

ANOVAs were conducted to determine if environmental conditions differed across BACI treatments to determine whether to include as potential explanatory variables in ANOVAs and GAMs.

**S3 Table. Short-term ANOVA mBACI Summary Statistics**

| | | ANOVA | | | |
|---|---|---|---|---|---|
| **Analysis** | **Response** | **Effect** | **DF** | **F** | **p** |
| **mBACI** | log(CPUE +1) ($km^{-1}$) | Period | 1, 161 | 4.935 | 0.028 |
| | | Year Type | 1, 161 | 0.214 | 0.644 |
| | | Period*Year Type | 1, 161 | 2.418 | 0.122 |
| | CPUE-Lr ($km^{-1}$) | Period | 1, 161 | 5.328 | 0.022 |
| | | Year Type | 1, 161 | 0.945 | 0.333 |
| | | Period*Year Type | 1, 161 | 2.591 | 0.109 |
| | Richness | Period | 1, 161 | 1.627 | 0.204 |
| | | Year Type | 1, 161 | 10.633 | 0.001 |
| | | Period*Year Type | 1, 161 | 2.970 | 0.087 |
| **Arthur BACI** | sqrt(CPUE) ($km^{-1}$) | Period | 1, 58 | 0.182 | 0.671 |
| | | Year Type | 1, 58 | 0.064 | 0.801 |
| | | Period*Year Type | 1, 58 | 0.21 | 0.210 |
| | sqrt(CPUE-Lr) ($km^{-1}$) | Period | 1, 58 | 0.28 | 0.599 |
| | | Year Type | 1, 58 | 8.57 | 0.005 |
| | | Period*Year Type | 1, 58 | 1.235 | 0.271 |
| | Richness | Period | 1, 58 | 0.626 | 0.432 |
| | | Year Type | 1, 58 | 1.867 | 0.177 |
| | | Period*Year Type | 1, 58 | 0.067 | 0.797 |

Summary results of short-term ANOVA tests across time period (before vs. after) and year type (control vs. impact year) for mBACI and Arthur BACI. Short-term analyses include only trawls conducted within 23 days of stormfall. Response variables were transformed when necessary to meet assumptions of parametric statistics. Post-hoc test results are not presented as no interaction term was significant.

**S4 Table. Mean values of CPUE, CPUE-Lr and Species Richness Across Treatments**

| | Time Frame | Year Type | Period | n | CPUE Mean ± SE | CPUE %Change* | CPUE-Lr Mean ± SE | CPUE-Lr %Change | Species Richness Mean ± SE | Species Richness %Change |
|---|---|---|---|---|---|---|---|---|---|---|
| mBACI | Short-term | Control | Before | 46 | 309 ± 65 | | 37 ± 9 | | 6.6 ± 0.4 | |
| | | Control | After | 33 | 182 ± 22 | -41.16 | 33 ± 5 | -10.78 | 6.9 ± 0.5 | 3.95 |
| | | Impact | Before | 53 | 261 ± 23 | | 50 ± 4 | | 8.5 ± 0.3 | |
| | | Impact | After | 33 | 151 ± 31 | -40.17 | 29 ± 3 | -43.03 | 7.5 ± 0.4 | -11.57 |
| | Seasonal | Control | Before | 239 | 376 ± 26 | | 46 ± 4 | | 6.1 ± 0.2 | |
| | | Control | After | 93 | 169 ± 18 | -55.02 | 23 ± 2 | -50 | 6.4 ± 0.3 | 4.72 |
| | | Impact | Before | 251 | 269 ± 13 | | 50 ± 3 | | 6.7 ± 0.2 | |
| | | Impact | After | 107 | 118 ± 15 | -56. | 18 ± 2 | -64.14 | 6.0 ± 0.3 | -9.323 |
| Arthur BACI | Short-term | Control | Before | 12 | 578 ± 90 | | 58 ± 9 | | 4.3 ± 0.5 | |
| | | Control | After | 12 | 402 ± 114 | -30.42 | 33 ± 7 | -42.27 | 4.8 ± 0.5 | 13.73 |
| | | Impact | Before | 12 | 498 ± 127 | | 87 ± 15 | | 7.9 ± 0.9 | |
| | | Impact | After | 26 | 582 ± 113 | 16.69 | 86 ± 14 | -1.14 | 7.5 ± 0.4 | -4.78 |
| | Seasonal | Control | Before | 23 | 507 ± 61 | | 61 ± 12 | | 4.0 ± 0.3 | |
| | | Control | After | 43 | 252 ± 46 | -50.35 | 26 ± 4 | -57.98 | 5.2 ± 0.4 | 30.65 |
| | | Impact | Before | 51 | 717 ± 67 | | 140 ± 12 | | 7.4 ± 0.3 | |
| | | Impact | After | 70 | 335 ± 50 | -53.37 | 56 ± 7 | -60.31 | 7.9 ± 0.4 | 7.15 |
| Matthew BACI | Seasonal | Control | Before | 48 | 94 ± 17 | | 16 ± 3 | | 4.9 ± 0.3 | |
| | | Control | After | 11 | 33 ± 9 | -64.74 | 5 ± 1 | -71.83 | 3.8 ± 0.4 | -21.68 |
| | | Impact | Before | 69 | 277 ± 29 | | 39 ± 4 | | 7.1 ± 0.4 | |
| | | Impact | After | 15 | 28 ± 5 | -89.97 | 10 ± 3 | -74.14 | 4.4 ± 0.4 | -38.42 |

Mean +/- standard error CPUE, CPUE-Lr, and species richness for multi-storm mBACI and BACI comparisons. Percent change is calculated as the decline or increase in catch or richness between before and after periods: $\frac{\text{before} - \text{after}}{\text{before}} \times 100$. **Note:** Means presented are rounded to whole values for catch and one decimal place for species richness. Percent change was calculated using unrounded values and may differ from calculations based upon means in the table.

**S5 Table. NMDS environmental correlates**

| | Short-term | | | Seasonal | | |
|---|---|---|---|---|---|---|
| **Factor** | **NMDS1** | **NMDS2** | **p** | **NMDS1** | **NMDS2** | **p** |
| Depth | -0.063 | 0.126 | 0.557 | 0.057 | -0.076 | 0.356 |
| Temperature | 0.012 | -0.394 | 0.007 | -0.021 | 0.143 | 0.084 |
| Salinity | 0.043 | -0.019 | 0.928 | -0.066 | -0.070 | 0.385 |
| Days since Storm | 0.147 | 0.134 | 0.284 | -0.240 | 0.554 | 0.001 |
| Storm Rainfall | -0.041 | 0.209 | 0.236 | 0.030 | -0.143 | 0.088 |
| Rainfall Anomaly | 0.104 | -0.136 | 0.417 | -0.043 | -0.028 | 0.746 |
| ACE | 0.005 | 0.166 | 0.419 | 0.011 | -0.147 | 0.085 |
| Winds | 0.121 | -0.042 | 0.608 | -0.022 | -0.081 | 0.456 |
| Gusts | 0.096 | 0.067 | 0.66 | -0.011 | -0.108 | 0.288 |
| Storm Surge | -0.018 | 0.191 | 0.315 | 0.020 | -0.151 | 0.064 |
| Antecedent Rain | 0.179 | 0.069 | 0.329 | -0.227 | 0.351 | 0.001 |

Summary results of environmental variables tested for potential correlation with fish community structure at the short-term and seasonal time frames.

**S6 Table. Contributing species to short-term and seasonal community group dissimilarities**

| | Scientific Name | Common Name | Avg. contrib to dissimilarity | Cum. contrib to dissimilarity |
|---|---|---|---|---|
| Short-term | **Before Control - After Control, Overall between-group dissimilarity = 0.4430** | | | |
| | *Lagadon rhomboides* (Linnaeus, 1766) | Pinfish | 0.0436 | 0.0931 |
| | *Gerreidae* spp. (Goode and Bean, 1879) | Mojarra spp. | 0.0435 | 0.1859 |
| | *Leiostomus xanthurus* (Lacepède, 1802) | Spot | 0.0416 | 0.2747 |
| | *Orthopristis chrysoptera* (Linnaeus, 1766) | Pigfish | 0.0409 | 0.3619 |
| | *Paralichthys* spp. (Jordan and Gilbert, 1882) | Flounder spp. | 0.0380 | 0.4429 |
| | *Bairdiella chrysoura* (Lacepède, 1802) | Silver Perch | 0.0344 | 0.5164 |
| | *Stephanolepis hispidus* (Linnaeus, 1766) | Planehead filefish | 0.0297 | 0.5798 |
| | *Lutjanus griseus* (Linneaus, 1758) | Grey Snapper | 0.0287 | 0.6409 |
| | *Lutjanus synagris* (Linneaus, 1758) | Lane Snapper | 0.0195 | 0.6825 |
| | *Anchoa* spp. (Valenciennes, 1848) | Anchovy spp. | 0.0172 | 0.7192 |
| | **Before Hurricane - After Hurricane, Overall between-group dissimilarity = 0.4498** | | | |
| | *Lagadon rhomboides* (Linnaeus, 1766) | Pinfish | 0.0417 | 0.0928 |
| | *Orthopristis chrysoptera* (Linnaeus, 1766) | Pigfish | 0.0378 | 0.1769 |
| | *Gerreidae* spp. (Goode and Bean, 1879) | Mojarra spp. | 0.0342 | 0.2529 |
| | *Stephanolepis hispidus* (Linnaeus, 1766) | Planehead filefish | 0.0339 | 0.3283 |
| | *Paralichthys* spp. (Jordan and Gilbert, 1882) | Flounder spp. | 0.0332 | 0.4022 |
| | *Bairdiella chrysoura* (Lacepède, 1802) | Silver Perch | 0.0330 | 0.4755 |
| | *Leiostomus xanthurus* (Lacepède, 1802) | Spot | 0.0324 | 0.5474 |
| | *Archosargus probatocephalus* (Walbaum, 1792) | Sheepshead | 0.0308 | 0.6160 |
| | *Lutjanus griseus* (Linneaus, 1758) | Grey Snapper | 0.0250 | 0.6717 |
| | *Sygnathus* spp. (Jordan and Gilbert 1882) | Pipefish spp. | 0.0184 | 0.7126 |
| | **Before Control - Before Hurricane, Overall between-group dissimilarity = 0.4437** | | | |
| | *Lagadon rhomboides* (Linnaeus, 1766) | Pinfish | 0.0499 | 0.1125 |
| | *Orthopristis chrysoptera* (Linnaeus, 1766) | Pigfish | 0.0381 | 0.1983 |
| | *Gerreidae* spp. (Goode and Bean, 1879) | Mojarra spp. | 0.0340 | 0.2749 |
| | *Leiostomus xanthurus* (Lacepède, 1802) | Spot | 0.0337 | 0.3509 |
| | *Stephanolepis hispidus* (Linnaeus, 1766) | Planehead filefish | 0.0337 | 0.4268 |
| | *Bairdiella chrysoura* (Lacepède, 1802) | Silver Perch | 0.0314 | 0.4974 |
| | *Archosargus probatocephalus* (Walbaum, 1792) | Sheepshead | 0.0313 | 0.5680 |
| | *Paralichthys* spp. (Jordan and Gilbert, 1882) | Flounder spp. | 0.0233 | 0.6205 |
| | *Diplodus holbrookii* (Bean, 1878) | Spottail pinfish | 0.0203 | 0.6663 |
| | *Sygnathus* spp. (Jordan and Gilbert 1882) | Pipefish spp. | 0.0178 | 0.7064 |

| | | | | |
|---|---|---|---|---|
| | **After Control - After Hurricane, Overall between-group dissimilarity = 0.4686** | | | |
| | *Lagadon rhomboides* (Linnaeus, 1766) | Pinfish | 0.0525 | 0.1187 |
| | *Gerreidae* spp. (Goode and Bean, 1879) | Mojarra spp. | 0.0447 | 0.2199 |
| | *Orthopristis chrysoptera* (Linnaeus, 1766) | Pigfish | 0.0393 | 0.3089 |
| | *Stephanolepis hispidus* (Linnaeus, 1766) | Planehead filefish | 0.0340 | 0.3858 |
| | *Leiostomus xanthurus* (Lacepède, 1802) | Spot | 0.0333 | 0.4610 |
| | *Paralichthys* spp. (Jordan and Gilbert, 1882) | Flounder spp. | 0.0271 | 0.5224 |
| | *Bairdiella chrysoura* (Lacepède, 1802) | Silver Perch | 0.0243 | 0.5774 |
| | *Lutjanus griseus* (Linneaus, 1758) | Grey Snapper | 0.0230 | 0.6294 |
| | *Diplodus holbrookii* (Bean, 1878) | Spottail pinfish | 0.0196 | 0.6738 |
| | *Mycteroperca microlepis* (Goode & Bean, 1879) | Gag grouper | 0.0140 | 0.7054 |
| **Long-term** | **Before Control - After Control, Overall between-group dissimilarity = 0.4826** | | | |
| | *Lagadon rhomboides* (Linnaeus, 1766) | Pinfish | 0.0674 | 0.1396 |
| | *Orthopristis chrysoptera* (Linnaeus, 1766) | Pigfish | 0.0539 | 0.2513 |
| | *Gerreidae* spp. (Goode and Bean, 1879) | Mojarra spp. | 0.0508 | 0.3567 |
| | *Leiostomus xanthurus* (Lacepède, 1802) | Spot | 0.0448 | 0.4496 |
| | *Paralichthys* spp. (Jordan and Gilbert, 1882) | Flounder spp. | 0.0347 | 0.5215 |
| | *Stephanolepis hispidus* (Linnaeus, 1766) | Planehead filefish | 0.0332 | 0.5903 |
| | *Bairdiella chrysoura* (Lacepède, 1802) | Silver Perch | 0.0240 | 0.6400 |
| | *Lutjanus griseus* (Linneaus, 1758) | Grey Snapper | 0.0220 | 0.6856 |
| | *Opsanus tau* (Linnaeus 1766) | Toadfish | 0.0176 | 0.7222 |
| | *Diplodus holbrookii* (Bean, 1878) | Spottail pinfish | 0.0158 | 0.7549 |
| | **Before Hurricane - After Hurricane, Overall between-group dissimilarity = 0.5023** | | | |
| | *Lagadon rhomboides* (Linnaeus, 1766) | Pinfish | 0.0636 | 0.1266 |
| | *Orthopristis chrysoptera* (Linnaeus, 1766) | Pigfish | 0.0610 | 0.2480 |
| | *Leiostomus xanthurus* (Lacepède, 1802) | Spot | 0.0473 | 0.3422 |
| | *Gerreidae* spp. (Goode and Bean, 1879) | Mojarra spp. | 0.0389 | 0.4197 |
| | *Paralichthys* spp. (Jordan and Gilbert, 1882) | Flounder spp. | 0.0376 | 0.4945 |
| | *Stephanolepis hispidus* (Linnaeus, 1766) | Planehead filefish | 0.0372 | 0.5685 |
| | *Bairdiella chrysoura* (Lacepède, 1802) | Silver Perch | 0.0306 | 0.6294 |
| | *Archosargus probatocephalus* (Walbaum, 1792) | Sheepshead | 0.0219 | 0.6731 |
| | *Diplodus holbrookii* (Bean, 1878) | Spottail pinfish | 0.0184 | 0.7098 |
| | *Lutjanus griseus* (Linneaus, 1758) | Grey Snapper | 0.0178 | 0.7452 |
| | **Before Control - Before Hurricane, Overall between-group dissimilarity = 0.4432** | | | |
| | *Lagadon rhomboides* (Linnaeus, 1766) | Pinfish | 0.0580 | 0.1309 |
| | *Orthopristis chrysoptera* (Linnaeus, 1766) | Pigfish | 0.0544 | 0.2537 |
| | *Leiostomus xanthurus* (Lacepède, 1802) | Spot | 0.0456 | 0.3566 |
| | *Stephanolepis hispidus* (Linnaeus, 1766) | Planehead filefish | 0.0358 | 0.4373 |
| | *Paralichthys* spp. (Jordan and Gilbert, 1882) | Flounder spp. | 0.0329 | 0.5114 |

| | | | | |
|---|---|---|---|---|
| | *Gerreidae* spp. (Goode and Bean, 1879) | Mojarra spp. | 0.0242 | 0.5659 |
| | *Bairdiella chrysoura* (Lacepède, 1802) | Silver Perch | 0.0239 | 0.6198 |
| | *Diplodus holbrookii* (Bean, 1878) | Spottail pinfish | 0.0206 | 0.6663 |
| | *Sygnathus* spp. (Jordan and Gilbert 1882) | Pipefish spp. | 0.0183 | 0.7077 |
| | *Opsanus tau* (Linnaeus 1766) | Toadfish | 0.0180 | 0.7482 |
| | **After Control - After Hurricane, Overall between-group dissimilarity = 0.4856** | | | |
| | *Lagadon rhomboides* (Linnaeus, 1766) | Pinfish | 0.0596 | 0.1228 |
| | *Orthopristis chrysoptera* (Linnaeus, 1766) | Pigfish | 0.0543 | 0.2346 |
| | *Gerreidae* spp. (Goode and Bean, 1879) | Mojarra spp. | 0.0471 | 0.3316 |
| | *Leiostomus xanthurus* (Lacepède, 1802) | Spot | 0.0426 | 0.4194 |
| | *Paralichthys* spp. (Jordan and Gilbert, 1882) | Flounder spp. | 0.0412 | 0.5044 |
| | *Bairdiella chrysoura* (Lacepède, 1802) | Silver Perch | 0.0314 | 0.5691 |
| | *Lutjanus griseus* (Linneaus, 1758) | Grey Snapper | 0.0296 | 0.6301 |
| | *Stephanolepis hispidus* (Linnaeus, 1766) | Planehead filefish | 0.0285 | 0.6888 |
| | *Lutjanus synagris* (Linneaus, 1758) | Lane Snapper | 0.0188 | 0.7274 |
| | *Archosargus probatocephalus* (Walbaum, 1792) | Sheepshead | 0.0158 | 0.7599 |

Results of Similarity Percentages (SIMPER) analysis indicating the species that contribute the most to dissimilarity across mBACI groups based on Bray-Curtis dissimilarities calculated from fourth-root transformed abundance data. Only the top 10 species that contribute the most to between-group dissimilarities are listed.

**S7 Table. Seasonal ANOVA and Tukey HSD for mBACI, Arthur- and Matthew BACIs**

| | | ANOVA | | | | Tukey HSD | |
|---|---|---|---|---|---|---|---|
| | **Response** | **Effect** | **DF** | **F** | **p** | **Comparison** | **p** |
| **mBACI** | CPUE $^{1/4}$ (km$^{-1}$) | Period | 1, 650 | 77.463 | <0.001 | | |
| | | Year Type | 1, 650 | 2.983 | 0.085 | | |
| | | Period*Year Type | 1, 650 | 2.069 | 0.151 | | |
| | CPUE-Lr $^{1/4}$ (km$^{-1}$) | Period | 1, 650 | 59.551 | <0.001 | CB-IB | 0.517 |
| | | Year Type | 1, 650 | 0.001 | 0.976 | CA-IA | 0.120 |
| | | Period*Year Type | 1, 650 | 6.802 | 0.009* | CB-CA & IB-IA | 0.002, <0.001 |
| | | | | | | CB-IA, IB-CA | <0.001, <0.001 |
| | Richness | Period | 1, 650 | 0.484 | 0.487 | CB-IB | 0.155 |
| | | Year Type | 1, 650 | 1.467 | 0.226 | CA-IA | 0.725 |
| | | Period*Year Type | 1, 650 | 4.016 | 0.046* | CB-CA & IB-IA | 0.798, 0.219 |
| | | | | | | CB-IA, IB-CA | 0.987, 0.907 |
| **Arthur BACI** | sqrt(CPUE) (km$^{-1}$) | Period | 1, 183 | 47.707 | <0.001 | | |
| | | Year Type | 1, 183 | 4.473 | 0.0358 | | |
| | | Period*Year Type | 1, 183 | 0.255 | 0.614 | | |
| | sqrt(CPUE-Lr) (km$^{-1}$) | Period | 1, 183 | 70.995 | <0.001 | | |
| | | Year Type | 1, 183 | 32.801 | <0.001 | | |
| | | Period*Year Type | 1, 183 | 3.379 | 0.068 | | |
| | Richness | Period | 1, 183 | 0.198 | 0.657 | | |
| | | Year Type | 1, 183 | 7.078 | 0.009 | | |
| | | Period*Year Type | 1, 183 | 0.418 | 0.519 | | |
| **Matthew BACI** | log(CPUE +1) (km$^{-1}$) | Period | 1, 139 | 50.137 | <0.001 | CB-IB | <0.001 |
| | | Year Type | 1, 139 | 30.256 | <0.001 | CA-IA | <0.001 |
| | | Period*Year Type | 1, 139 | 6.689 | 0.011* | CB-CA & IB-IA | 0.102, <0.001 |
| | | | | | | CB-IA, IB-CA | 0.880, <0.001 |
| | log(CPUE-Lr +1) (km$^{-1}$) | Period | 1, 139 | 22.756 | <0.001 | | |
| | | Year Type | 1, 139 | 25.473 | <0.001 | | |
| | | Period*Year Type | 1, 139 | 0.828 | 0.364 | | |
| | sqrt(Richness) (km$^{-1}$) | Period | 1, 139 | 14.66 | <0.001 | | |
| | | Year Type | 1, 139 | 21.04 | <0.001 | | |
| | | Period*Year Type | 1, 139 | 2.25 | 0.136 | | |

Summary results of seasonal-scale ANOVA tests across time period (before vs. after) and year type (control vs. impact year). Seasonal analyses include trawls conducted during the months of May-October. Response variables were transformed when necessary to meet assumptions of parametric statistics. Post-hoc test results are presented when the interaction term is significant at p<0.05. Abbreviations indicate treatments; CB = control before, IB = impact before, CA = control after, IA = impact after

**S8 Table. GAM Model Summary Statistics for Seasonal mBACI and Hurricane Arthur**

| | | | edf | p | logLik | Dev | df.r | AIC | BIC |
|---|---|---|---|---|---|---|---|---|---|
| **Season** | **CPUE** | Control : Before | 0.901 | 0.044 | -2201.3 | 280.2 | 237.1 | 4409.1 | 4420.3 |
| | | Impact : Before | 0.023 | 0.356 | -2217.0 | 273.8 | 250.0 | 4438.1 | 4445.4 |
| | | Control : After | 1.785 | <0.001 | -773.7 | 103.0 | 90.2 | 1555.4 | 1565.4 |
| | | Impact : After | 1.708 | <0.001 | -846.4 | 119.5 | 104.3 | 1700.6 | 1711.0 |
| | **CPUE-Lr** | Control : Before | 0.637 | 0.129 | -1703.6 | 278.3 | 237.4 | 3413.0 | 3423.3 |
| | | Impact : Before | 0.003 | 0.426 | -1817.2 | 290.9 | 250.0 | 3638.4 | 3645.5 |
| | | Control : After | 1.672 | <0.001 | -582.9 | 99.4 | 90.3 | 1173.5 | 1183.2 |
| | | Impact : After | 1.545 | <0.001 | -653.7 | 126.3 | 104.5 | 1314.8 | 1324.7 |
| | **Richness** | Control : Before | <0.001 | 0.466 | -539.6 | 218.1 | 238.0 | 1083.2 | 1090.2 |
| | | Impact : Before | 1.755 | <0.001 | -559.8 | 199.1 | 248.2 | 1127.4 | 1141.0 |
| | | Control : After | 1.437 | 0.001 | -220.1 | 89.9 | 90.6 | 447.8 | 457.3 |
| | | Impact : After | 1.711 | <0.001 | -241.0 | 106.7 | 104.3 | 489.8 | 500.1 |
| **Arthur** | **CPUE** | Control : Before | 0.003 | 0.326 | -162.5 | 23.7 | 22.0 | 329.0 | 331.3 |
| | | Impact : Before | 0.993 | 0.02 | -378.2 | 53.0 | 49.0 | 763.1 | 769.5 |
| | | Control : After | 0.908 | 0.037 | -277.5 | 49.2 | 41.1 | 561.6 | 567.3 |
| | | Impact : After | 1.803 | <0.001 | -456.5 | 74.6 | 67.2 | 920.7 | 929.4 |
| | **CPUE-Lr** | Control : Before | <0.001 | 0.764 | -117.1 | 25.0 | 22.0 | 238.3 | 240.6 |
| | | Impact : Before | 1.684 | 0.005 | -291.8 | 53.2 | 48.3 | 591.5 | 599.1 |
| | | Control : After | 1.55 | 0.001 | -178.4 | 44.9 | 40.5 | 364.4 | 371.1 |
| | | Impact : After | 1.739 | <0.001 | -336.8 | 75.4 | 67.3 | 681.4 | 690.2 |
| | **Richness** | Control : Before | 0.598 | 0.116 | -49.4 | 13.7 | 21.4 | 104.4 | 107.6 |
| | | Impact : Before | <0.001 | 0.47 | -115.2 | 35.7 | 50.0 | 234.4 | 238.2 |
| | | Control : After | <0.001 | 0.467 | -105.7 | 41.5 | 42.0 | 215.3 | 218.9 |
| | | Impact : After | <0.001 | 0.708 | -182.3 | 69.3 | 69.0 | 368.7 | 373.2 |

All GAM models were run for the seasonal time frame against days since storm as the independent variable and built using a cubic regression spline with penalized shrinkage, a maximum of three degrees of freedoms, negative binomial error distribution with log link function, and restricted maximum likelihood smoothing parameter. edf = effective degrees of freedom, logLik = log likelihood, Dev = deviance, df.r = residual degrees of freedom, AIC = Akaike information criterion BIC = Bayesian information criterion.

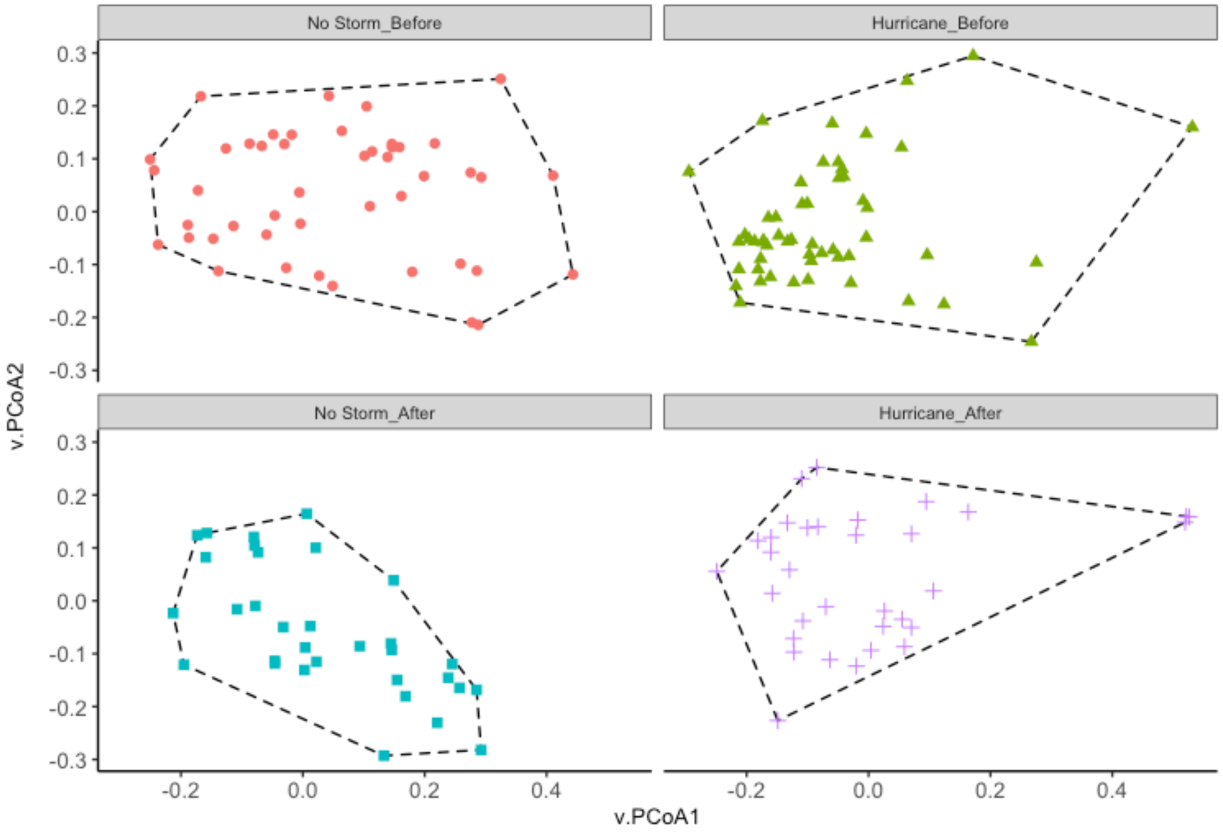


**S2 Figure. Individual mBACI treatment PCoA ordinations of short-term communities**
This figure demonstrates the potential difference/lack of difference in short-term community dispersion within each mBACI treatment using Principle Coordinates Analysis. Convex hulls are drawn in dashed lines through the outer-must points.

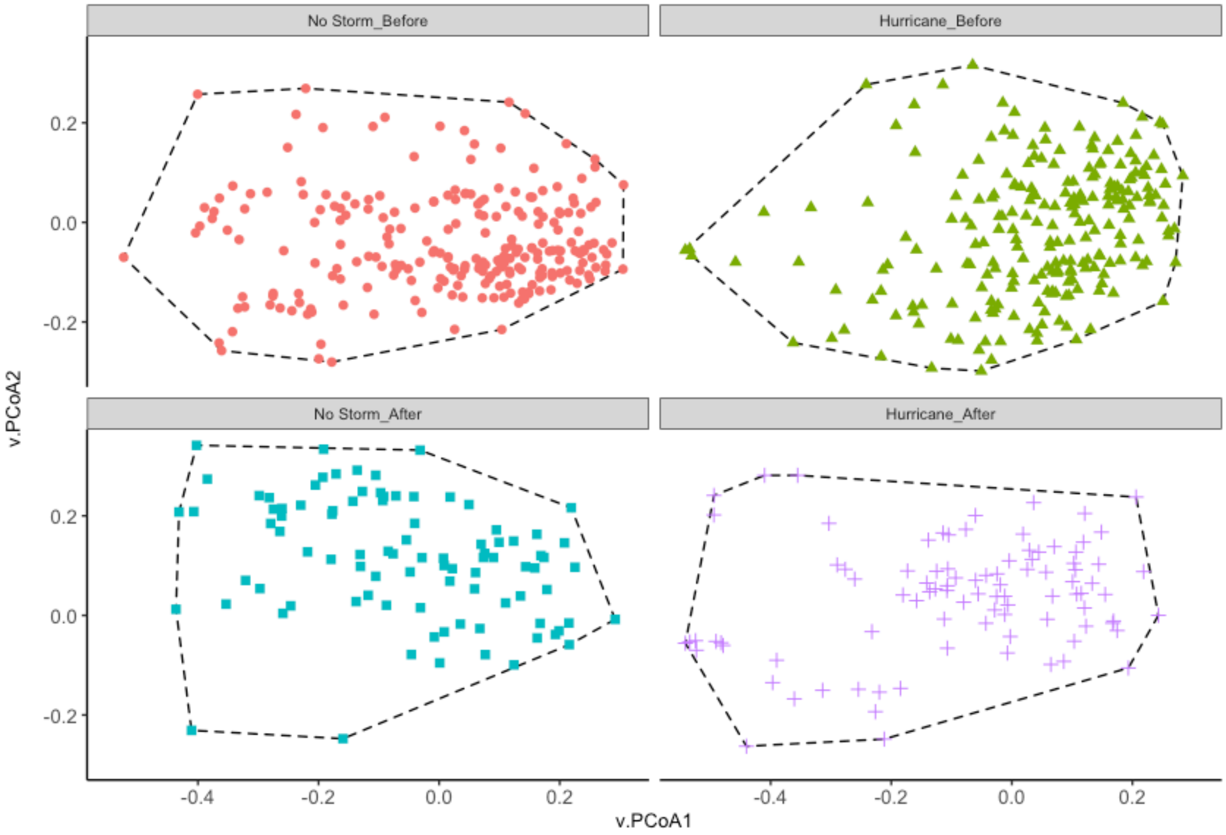


**S3 Figure. Individual mBACI group PCoA ordinations of seasonal communities**
This figure demonstrates the potential difference/lack of difference in seasonal community dispersion within each mBACI treatment using Principle Coordinates Analysis. Convex hulls are drawn in dashed lines through the outer-must points.

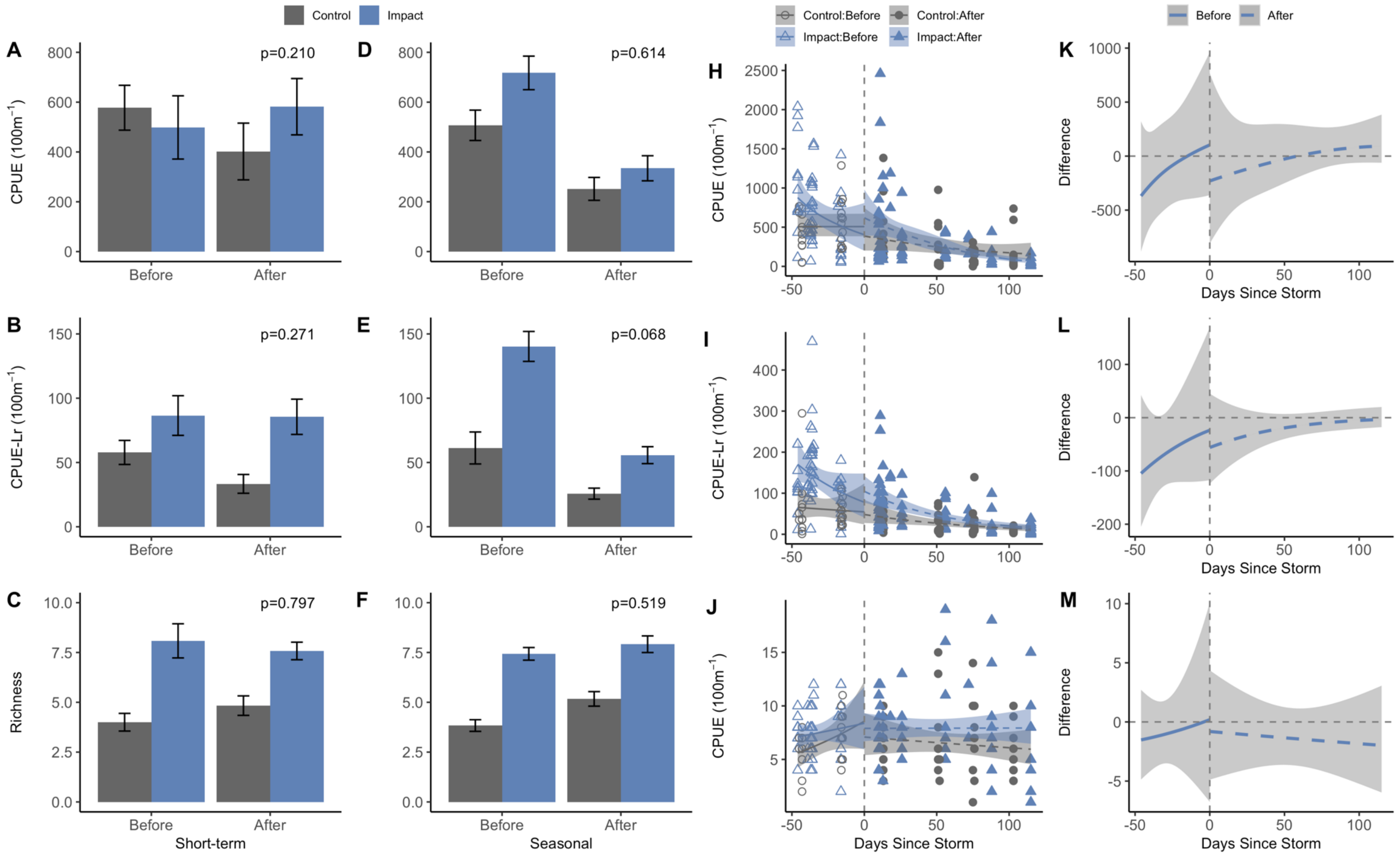


**S4 Figure.** Short-term and seasonal fish catches and species richness across time periods and year type for Hurricane Arthur (July 2014) compared to 2015 (control year). Only means are presented for short-term comparisons (column 1); whereas means, trend, and difference between control and impact trends (columns 2-4, respectively) are depicted for seasonal comparisons. Catch per unit effort (CPUE) is presented in row 1; CPUE calculated sans *L. rhomboides* is presented in row 2, and species richness is row 3. P-values indicate the significance of the interactive ANOVA term. Error bars represent standard error. Smoothed lines represent generalized additive models (y ~ s(Days to Storm), k = 3) for both hurricane and storm-free years based on a cubic regression spline with shrinkage and 95% confidence intervals.

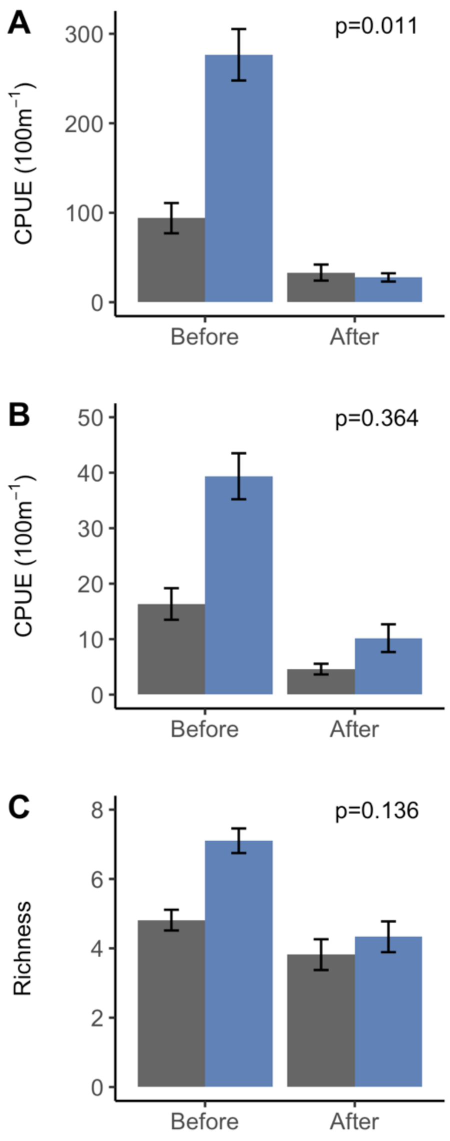


**S5 Figure.** Seasonal fish catch per unit effort (A), catch per unit effort sans *Lagodon rhomboides* (B) and species richness (C) across time periods (before vs. after) and year type (control vs. impact) for Hurricane Matthew (October 2016) compared to 2017 (control year). Only means are presented for seasonal comparisons (column 1), as the closest trawl samples prior to hurricane Matthew occurred outside of the short-term window and all trawls that occurred after the storm were conducted on the same day. P-values indicate the significance of the interactive ANOVA term. Error bars represent standard error.
