# Supplementary material for: Tropical cyclone impacts on seagrass-associated fishes in a temperate-subtropical estuary
Source: PLoS One. 2022 Oct 13;17(10):e0273556. doi: 10.1371/journal.pone.0273556 (PMC9560482; doi:10.1371/journal.pone.0273556)
Supplement: S7 Table — ANOVA results for seasonal time frame analyses conducted for multi-storm mBACI, Arthur BACI, and Matthew BACI. Post-hoc test results are presented when the interaction term is significant at p<0.05. (PDF) [file pone.0273556.s008.pdf]

Supplementary Information

S1 Appendix. Literature Review Citations for Table 1
Published studies that examined change in fish abundances before and after hurricanes listed in the literature review.

S1 Figure. Statistical Analysis Flowchart and Transformations
Decision-tree indicating how fish data was subsetted, transformed, and analyzed. If not listed as transformed, raw data was used as the response variable.

S1 Table. Seagrass meadow locations and survey dates
Meadows surveyed for both fishes and percent cover of seagrass. Seagrass survey dates (month/year) and periodicity listed.

S2 Table. Temperature and Salinity ANOVA summary statistics
Summary results examining potential correlations between environmental variables, namely surface water temperature and salinity, fish catches and species richness.

S3 Table. Short-term ANOVA summary statistics
Summary results of short-term ANOVA tests across time period (before vs. after) and year type (control vs. impact year) for mBACI and Arthur BACI. Short-term analyses include only trawls conducted within 23 days of stormfall. Response variables were transformed when necessary to meet assumptions of parametric statistics. Post-hoc test results are not presented as no interaction term was significant.

S4 Table. Mean fish metrics across mBACI treatments
Mean +/- standard error CPUE, CPUE-Lr, and species richness for multi-storm mBACI and BACI comparisons. Percent change is calculated as the decline or increase in catch or richness between before and after periods: $\frac{\text{before} - \text{after}}{\text{before}} \times 100$. **Note:** Means presented are rounded to whole values for catch and one decimal place for species richness. Percent change was calculated using unrounded values and may differ from calculations based upon means in the table.

S5 Table. NMDS environmental correlates
Summary results of environmental variables tested for potential correlation with fish community structure at the short-term and seasonal time frames.

S6 Table. Contributing species to group dissimilarities
Results of Similarity Percentages (SIMPER) analysis indicating the species that contribute the most to dissimilarity across BACI groups based on Bray-Curtis dissimilarities calculated from fourth-root transformed abundance data. Only the top 10 species that contribute the most to between-group dissimilarities are listed.

S7 Table. Seasonal ANOVA and Tukey HSD for mBACI, Arthur- and Matthew BACIs
Summary results of seasonal-scale ANOVA tests across time period (before vs. after) and year type (control vs. impact year). Seasonal analyses include trawls conducted during the months of

May-October. Response variables were transformed when necessary to meet assumptions of parametric statistics. Post-hoc test results are presented when the interaction term is significant at $p<0.05$. Abbreviations indicate treatments; CB = control before, IB = impact before, CA = control after, IA = impact after

S8 Table. GAM Summary Statistics for Seasonal mBACI
All generalized additive models (GAMs) were run for the seasonal time frame against days since storm as the independent variable and built using a cubic regression spline with penalized shrinkage, a maximum of three degrees of freedoms, negative binomial error distribution with log link function, and restricted maximum likelihood smoothing parameter. edf = effective degrees of freedom, logLik = log likelihood, Dev = deviance, df.r = residual degrees of freedom, AIC = Akaike information criterion BIC = Bayesian information criterion.

S9 Table. Hurricane Arthur GLM Summary Statistics
Negative binomial generalized linear models for seasonal-scale trend analysis of Hurricane Arthur (2014) versus 2015 as the control year. Est = estimate, df.r = residual degrees of freedom, AIC = Akaike information criterion BIC = Bayesian information criterion.

S2 Figure. Individual mBACI treatment PCoA ordinations of short-term communities
This figure demonstrates the potential difference/lack of difference in short-term community dispersion within each mBACI treatment using Principle Coordinates Analysis. Convex hulls are drawn in dashed lines through the outer-must points.

S3 Figure. Individual mBACI group PCoA ordinations of seasonal communities
This figure demonstrates the potential difference/lack of difference in seasonal community dispersion within each mBACI treatment using Principle Coordinates Analysis. Convex hulls are drawn in dashed lines through the outer-must points.

S4 Figure. Hurricane Arthur BACI comparisons
Short-term and seasonal fish catches and species richness across time periods (before vs. after) and year type (control vs. impact) for Hurricane Arthur (July 2014) compared to 2015 (control year). Only means are presented for short-term comparisons (column 1); whereas means, trend, and difference between control and impact trends (columns 2-4, respectively) are depicted for seasonal comparisons. Catch per unit effort (CPUE) is presented in row 1 (A, D, H, K); CPUE calculated sans *L. rhomboides* is presented in row 2 (B, E. I, L), and species richness is row 3 (C, F, J, M). P-values indicate the significance of the interactive ANOVA term. Error bars represent standard error. Smoothed lines represent generalized additive models (y ~ s(Days to Storm), k = 3) for both hurricane and storm-free years based on a cubic regression spline with shrinkage and 95% confidence intervals.

S5 Figure. Hurricane Matthew Seasonal BACI comparisons
Seasonal fish catch per unit effort (A), catch per unit effort sans *Lagodon rhomboides* (B) and species richness (C) across time periods (before vs. after) and year type (control vs. impact) for Hurricane Matthew (October 2016) compared to 2017 (control year). Only means are presented for seasonal comparisons (column 1), as the closest trawl samples prior to hurricane Matthew occurred outside of the short-term window and all trawls that occurred after the storm were

conducted on the same day. P-values indicate the significance of the interactive ANOVA term. Error bars represent standard error.

S1 Appendix. Literature Review Citations for Table 1

Published studies that examined change in fish abundances before and after hurricanes listed in the literature review.

1. Adams A. Effects of a hurricane on two assemblages of coral reef fishes: Multiple-year analysis reverses a false "snapshot" interpretation. Bull Mar Sci. 2001;69: 341–356.
2. Adams AJ, Ebersole JP. Resistance of coral reef fishes in back reef and lagoon habitats to a hurricane. Bull Mar Sci. 2004;75: 101–113.
3. Anton A, Cebrian J, Duarte CM, Heck Jr KL, Goff J. Low impact of Hurricane Katrina on seagrass community structure and functioning in the northern Gulf of Mexico. Bull Mar Sci. 2009;85: 45–59.
4. Bortone SA. Effects of a hurricane on the fish fauna at Destin, Florida. Flor Sci. 1976; 245–248.
5. Bouchon C, Bouchon-Navaro Y, Louis M. Changes in the coastal fish communities following hurricane Hugo in Guadelope Island (French West Indies). Atoll Res Bull. 1994.
6. Burkholder J, Eggleston D, Glasgow H, Brownie C, Reed R, Janowitz G, et al. Comparative impacts of two major hurricane seasons on the Neuse River and western Pamlico Sound ecosystems. Proc Natl Acad Sci USA. 2004;101: 9291–9296.
7. Cheal A, Coleman G, Delean S, Miller I, Osborne K, Sweatman H. Responses of coral and fish assemblages to a severe but short-lived tropical cyclone on the Great Barrier Reef, Australia. Coral Reefs. 2002;21: 131–142.
8. Davis J, Laird B. The effects of tropical storm Agnes on the Chesapeake Bay estuarine system. The Chesapeake Reserach Consortium; 1976. Report No.: CRC Publication No. 54.
9. Dolloff CA, Flebbe PA, Owen MD. Fish habitat and fish populations in a southern Appalachian watershed before and after Hurricane Hugo. Trans Am Fish Soc. 1994;123: 668–678.
10. Fenner DP. Effects of Hurricane Gilbert on Coral Reefs, Fishes and Sponges at Cozumel, Mexico. Bull Mar Sci. 1991;48: 719–730.
11. Fitzsimons JM, Nishimoto RT. Use of fish behavior in assessing the effects of Hurricane Iniki on the Hawaiian island of Kaua'i. Environ Biol Fishes. 1995;43: 39–50.
12. Greenwood MF, Stevens PW, Matheson RE. Effects of the 2004 hurricanes on the fish assemblages in two proximate southwest Florida estuaries: change in the context of interannual variability. Estuaries Coast. 2006;29: 985–996.
13. Lassig BR. The effects of a cyclonic storm on coral reef fish assemblages. Environ Biol Fishes. 1983;9: 55–63.
14. Letourneur Y, Harmelin-Vivien M, Galzin R. Impact of hurricane Firinga on fish community structure on fringing reefs of Reunion Island, S.W. Indian Ocean. Environ Biol Fishes. 1993;37: 109–120. doi:10.1007/BF00000586
15. Locascio JV, Mann DA. Effects of Hurricane Charley on fish chorusing. Biol Lett. 2005;1: 362–365.

16. Paerl HW, Bales JD, Ausley LW, Buzzelli CP, Crowder LB, Eby LA, et al. Ecosystem impacts of three sequential hurricanes (Dennis, Floyd, and Irene) on the United States' largest lagoonal estuary, Pamlico Sound, NC. Proc Natl Acad Sci USA. 2001;98: 5655–5660.

17. Paperno R, Tremain D, Adams D, Sebastian A, Sauer J, Dutka-Gianelli J. The disruption and recovery of fish communities in the Indian River Lagoon, Florida, following two hurricanes in 2004. Estuaries Coast. 2006;29: 1004–1010.

18. Springer VG, McErlean AJ. A study of the behavior of some tagged south Florida coral reef fishes. Am Midl Nat. 1962; 386–397.

19. Stevens PW, Blewett DA, Casey JP. Short-term effects of a low dissolved oxygen event on estuarine fish assemblages following the passage of Hurricane Charley. Estuaries Coast. 2006;29: 997–1003.

20. Switzer T, Winner B, Dunham N, Whittington J, Thomas M. Influence of sequential hurricanes on nekton communities in a southeast Florida estuary: short-term effects in the context of historical variations in freshwater inflow. Estuaries Coast. 2006;29: 1011–1018.

21. Yu J, Tang D, Chen G, Li Y, Huang Z, Wang S. The positive effects of typhoons on the fish CPUE in the South China Sea. Cont Shelf Res. 2014;84: 1–12. doi:10.1016/j.csr.2014.04.025

22. Yu J, Tang D, Li Y, Huang Z, Chen G. Increase in fish abundance during two typhoons in the South China Sea. Adv Space Res. 2013;51: 1734–1749. doi:10.1016/j.asr.2012.11.019

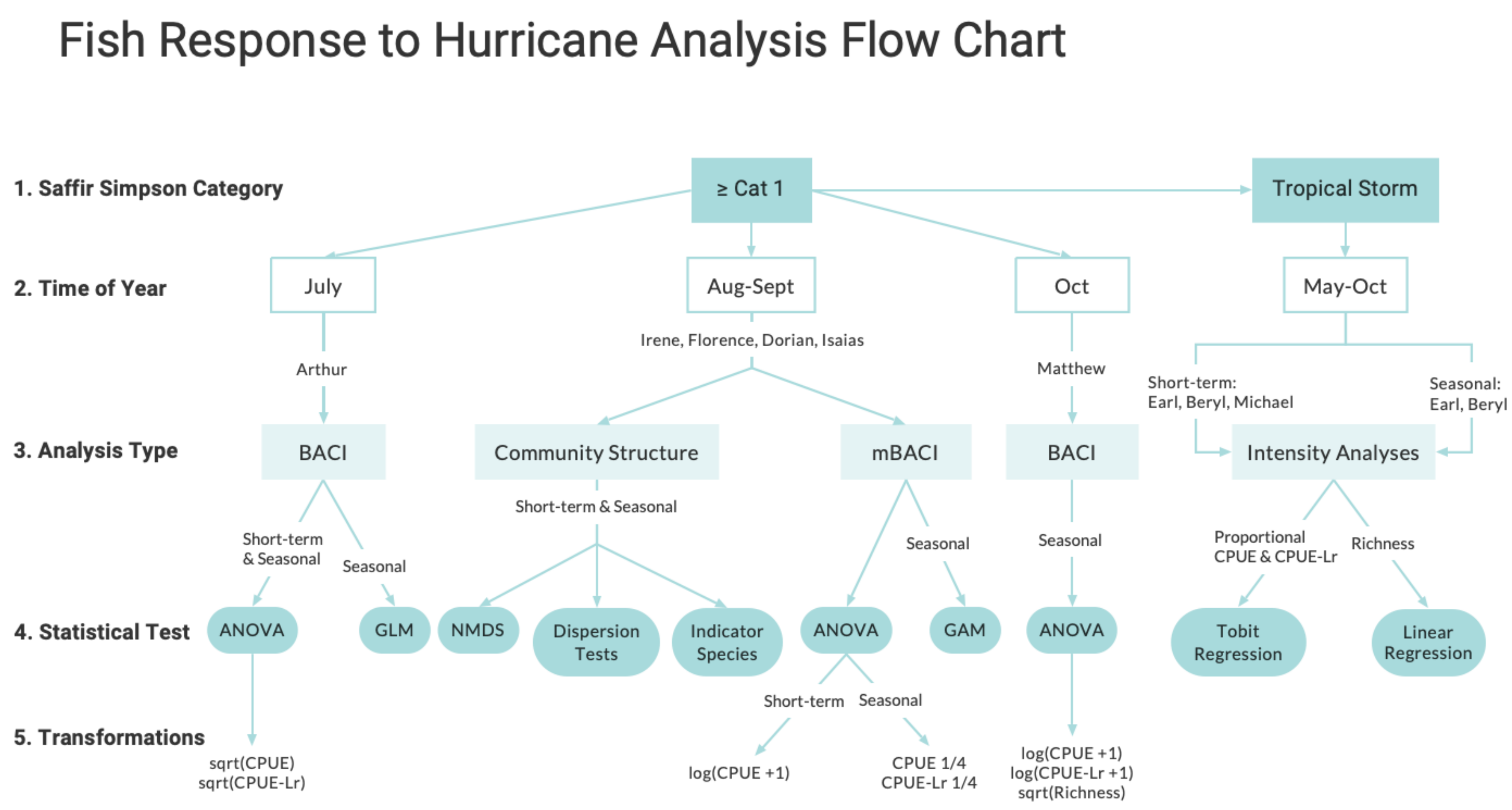

S1 Figure. Statistical Analysis Flowchart and Transformations
Decision-tree indicating how fish data was subsetted, transformed, and analyzed. If not listed as transformed, raw data was used as the response variable.

S1 Table. Seagrass meadow locations and survey dates

Meadow	Latitude	Longitude	Dominant Seagrass spp.	Surveys
SG 1	34.691747	-76.622573	*Zostera marina*	May-Oct 2016 (monthly)<br>Apr-Sep 2019 (monthly)
SG 2	34.697874	-76.595503	*Halodule wrightii*	Aug & Oct 2013<br>May & July 2014<br>May-Oct 2016 (monthly)<br>Apr-Sep 2019 (monthly)
SG 3	34.699899	-76.592917	*Halodule wrightii*	Aug & Oct 2013<br>May & July 2014<br>May-Oct 2016 (monthly)<br>Apr-Sep 2019 (monthly)
SG 4	34.703403	-76.587869	*Halodule wrightii*	May-Oct 2016 (monthly)<br>Apr-Sep 2019 (monthly)

Meadows surveyed for both fishes and percent cover of seagrass. Seagrass survey dates (month/year) and periodicity listed.

S2 Table. Temperature and Salinity ANOVA summary statistics

	Time Frame	Factor	Df	Sum Sq	Mean Sq	F value	P
**Temperature**	**Short-term**	Period	1	26.3	26.28	1.741	0.189
		Year Type	1	18	19.98	1.191	0.277
		Period*Year Type	1	24.9	24.94	1.652	0.201
		Residuals	150	2264.6	15.1		
	**Seasonal**	Period	1	54	53.83	3.775	0.052
		Year Type	1	45	45.31	3.178	0.075
		Period*Year Type	1	25	24.51	1.719	0.190
		Residuals	603	8597	14.26		
**Salinity**	**Short-term**	Period	1	2.3	2.267	0.493	0.484
		Year Type	1	6.2	6.16	1.341	0.249
		Period*Year Type	1	0.3	0.321	0.1	0.792
		Residuals	142	652.4	4.594		
	**Seasonal**	Period	1	0	0.196	0.031	0.859
		Year Type	1	7	7.191	1.154	0.283
		Period*Year Type	1	22	22.286	3.575	0.060
		Residuals	549	3422	6.234		

ANOVAs were conducted to determine if environmental conditions differed across BACI treatments to determine whether to include as potential explanatory variables in ANOVAs and GAMs.

S3 Table. Short-term ANOVA Summary Statistics

		ANOVA			
**Analysis**	**Response**	**Effect**	**DF**	**F**	**p**
**mBACI**	log(CPUE +1) (km^{-1})	Period	1, 161	4.935	0.028
		Year Type	1, 161	0.214	0.644
		Period*Year Type	1, 161	2.418	0.122
	CPUE-Lr (km^{-1})	Period	1, 161	5.328	0.022
		Year Type	1, 161	0.945	0.333
		Period*Year Type	1, 161	2.591	0.109
	Richness	Period	1, 161	1.627	0.204
		Year Type	1, 161	10.633	0.001
		Period*Year Type	1, 161	2.970	0.087
**Arthur BACI**	sqrt(CPUE) (km^{-1})	Period	1, 58	0.182	0.671
		Year Type	1, 58	0.064	0.801
		Period*Year Type	1, 58	0.21	0.210
	sqrt(CPUE-Lr) (km^{-1})	Period	1, 58	0.28	0.599
		Year Type	1, 58	8.57	0.005
		Period*Year Type	1, 58	1.235	0.271
	Richness	Period	1, 58	0.626	0.432
		Year Type	1, 58	1.867	0.177
		Period*Year Type	1, 58	0.067	0.797

Summary results of short-term ANOVA tests across time period (before vs. after) and year type (control vs. impact year) for mBACI and Arthur BACI. Short-term analyses include only trawls conducted within 23 days of stormfall. Response variables were transformed when necessary to meet assumptions of parametric statistics. Post-hoc test results are not presented as no interaction term was significant.

S4 Table. Mean values of CPUE, CPUE-Lr and Species Richness Across Treatments

	Time Frame	Year Type	Period	n	CPUE Mean ± SE	CPUE %Change*	CPUE-Lr Mean ± SE	CPUE-Lr %Change	Species Richness Mean ± SE	Species Richness %Change
mBACI	Short-term	Control	Before	46	309 ± 65		37 ± 9		6.6 ± 0.4	
		Control	After	33	182 ± 22	-41.16	33 ± 5	-10.78	6.9 ± 0.5	3.95
		Impact	Before	53	261 ± 23		50 ± 4		8.5 ± 0.3	
		Impact	After	33	151 ± 31	-40.17	29 ± 3	-43.03	7.5 ± 0.4	-11.57
	Seasonal	Control	Before	239	376 ± 26		46 ± 4		6.1 ± 0.2	
		Control	After	93	169 ± 18	-55.02	23 ± 2	-50	6.4 ± 0.3	4.72
		Impact	Before	251	269 ± 13		50 ± 3		6.7 ± 0.2	
		Impact	After	107	118 ± 15	-56.	18 ± 2	-64.14	6.0 ± 0.3	-9.323
Arthur BACI	Short-term	Control	Before	12	578 ± 90		58 ± 9		4.3 ± 0.5	
		Control	After	12	402 ± 114	-30.42	33 ± 7	-42.27	4.8 ± 0.5	13.73
		Impact	Before	12	498 ± 127		87 ± 15		7.9 ± 0.9	
		Impact	After	26	582 ± 113	16.69	86 ± 14	-1.14	7.5 ± 0.4	-4.78
	Seasonal	Control	Before	23	507 ± 61		61 ± 12		4.0 ± 0.3	
		Control	After	43	252 ± 46	-50.35	26 ± 4	-57.98	5.2 ± 0.4	30.65
		Impact	Before	51	717 ± 67		140 ± 12		7.4 ± 0.3	
		Impact	After	70	335 ± 50	-53.37	56 ± 7	-60.31	7.9 ± 0.4	7.15
Matthew BACI	Seasonal	Control	Before	48	94 ± 17		16 ± 3		4.9 ± 0.3	
		Control	After	11	33 ± 9	-64.74	5 ± 1	-71.83	3.8 ± 0.4	-21.68
		Impact	Before	69	277 ± 29		39 ± 4		7.1 ± 0.4	
		Impact	After	15	28 ± 5	-89.97	10 ± 3	-74.14	4.4 ± 0.4	-38.42

Mean +/- standard error CPUE, CPUE-Lr, and species richness for multi-storm mBACI and BACI comparisons. Percent change is calculated as the decline or increase in catch or richness between before and after periods: $\frac{\text{before} - \text{after}}{\text{before}} \times 100$. **Note:** Means presented are rounded to whole values for catch and one decimal place for species richness. Percent change was calculated using unrounded values and may differ from calculations based upon means in the table.

S5 Table. NMDS environmental correlates

	Short-term			Seasonal		
**Factor**	**NMDS1**	**NMDS2**	**p**	**NMDS1**	**NMDS2**	**p**
Depth	-0.063	0.126	0.557	0.057	-0.076	0.356
Temperature	0.012	-0.394	0.007	-0.021	0.143	0.084
Salinity	0.043	-0.019	0.928	-0.066	-0.070	0.385
Days since Storm	0.147	0.134	0.284	-0.240	0.554	0.001
Storm Rainfall	-0.041	0.209	0.236	0.030	-0.143	0.088
Rainfall Anomaly	0.104	-0.136	0.417	-0.043	-0.028	0.746
ACE	0.005	0.166	0.419	0.011	-0.147	0.085
Winds	0.121	-0.042	0.608	-0.022	-0.081	0.456
Gusts	0.096	0.067	0.66	-0.011	-0.108	0.288
Storm Surge	-0.018	0.191	0.315	0.020	-0.151	0.064
Antecedent Rain	0.179	0.069	0.329	-0.227	0.351	0.001

Summary results of environmental variables tested for potential correlation with fish community structure at the short-term and seasonal time frames.

S6 Table. Contributing species to short-term and seasonal community group dissimilarities

	Scientific Name	Common Name	Avg. contrib to dissimilarity	Cum. contrib to dissimilarity
Short-term	**Before Control - After Control, Overall between-group dissimilarity = 0.4430**			
	Lagadon rhomboides (Linnaeus, 1766)	Pinfish	0.0436	0.0931
	Gerreidae spp. (Goode and Bean, 1879)	Mojarra spp.	0.0435	0.1859
	Leiostomus xanthurus (Lacepède, 1802)	Spot	0.0416	0.2747
	Orthopristis chrysoptera (Linnaeus, 1766)	Pigfish	0.0409	0.3619
	Paralichthys spp. (Jordan and Gilbert, 1882)	Flounder spp.	0.0380	0.4429
	Bairdiella chrysoura (Lacepède, 1802)	Silver Perch	0.0344	0.5164
	Stephanolepis hispidus (Linnaeus, 1766)	Planehead filefish	0.0297	0.5798
	Lutjanus griseus (Linneaus, 1758)	Grey Snapper	0.0287	0.6409
	Lutjanus synagris (Linneaus, 1758)	Lane Snapper	0.0195	0.6825
	Anchoa spp. (Valenciennes, 1848)	Anchovy spp.	0.0172	0.7192
	**Before Hurricane - After Hurricane, Overall between-group dissimilarity = 0.4498**			
	Lagadon rhomboides (Linnaeus, 1766)	Pinfish	0.0417	0.0928
	Orthopristis chrysoptera (Linnaeus, 1766)	Pigfish	0.0378	0.1769
	Gerreidae spp. (Goode and Bean, 1879)	Mojarra spp.	0.0342	0.2529
	Stephanolepis hispidus (Linnaeus, 1766)	Planehead filefish	0.0339	0.3283
	Paralichthys spp. (Jordan and Gilbert, 1882)	Flounder spp.	0.0332	0.4022
	Bairdiella chrysoura (Lacepède, 1802)	Silver Perch	0.0330	0.4755
	Leiostomus xanthurus (Lacepède, 1802)	Spot	0.0324	0.5474
	Archosargus probatocephalus (Walbaum, 1792)	Sheepshead	0.0308	0.6160
	Lutjanus griseus (Linneaus, 1758)	Grey Snapper	0.0250	0.6717
	Sygnathus spp. (Jordan and Gilbert 1882)	Pipefish spp.	0.0184	0.7126
	**Before Control - Before Hurricane, Overall between-group dissimilarity = 0.4437**			
	Lagadon rhomboides (Linnaeus, 1766)	Pinfish	0.0499	0.1125
	Orthopristis chrysoptera (Linnaeus, 1766)	Pigfish	0.0381	0.1983
	Gerreidae spp. (Goode and Bean, 1879)	Mojarra spp.	0.0340	0.2749
	Leiostomus xanthurus (Lacepède, 1802)	Spot	0.0337	0.3509
	Stephanolepis hispidus (Linnaeus, 1766)	Planehead filefish	0.0337	0.4268
	Bairdiella chrysoura (Lacepède, 1802)	Silver Perch	0.0314	0.4974
	Archosargus probatocephalus (Walbaum, 1792)	Sheepshead	0.0313	0.5680
	Paralichthys spp. (Jordan and Gilbert, 1882)	Flounder spp.	0.0233	0.6205
	Diplodus holbrookii (Bean, 1878)	Spottail pinfish	0.0203	0.6663
	Sygnathus spp. (Jordan and Gilbert 1882)	Pipefish spp.	0.0178	0.7064

	**After Control - After Hurricane, Overall between-group dissimilarity = 0.4686**			
	Lagadon rhomboides (Linnaeus, 1766)	Pinfish	0.0525	0.1187
	Gerreidae spp. (Goode and Bean, 1879)	Mojarra spp.	0.0447	0.2199
	Orthopristis chrysoptera (Linnaeus, 1766)	Pigfish	0.0393	0.3089
	Stephanolepis hispidus (Linnaeus, 1766)	Planehead filefish	0.0340	0.3858
	Leiostomus xanthurus (Lacepède, 1802)	Spot	0.0333	0.4610
	Paralichthys spp. (Jordan and Gilbert, 1882)	Flounder spp.	0.0271	0.5224
	Bairdiella chrysoura (Lacepède, 1802)	Silver Perch	0.0243	0.5774
	Lutjanus griseus (Linneaus, 1758)	Grey Snapper	0.0230	0.6294
	Diplodus holbrookii (Bean, 1878)	Spottail pinfish	0.0196	0.6738
	Mycteroperca microlepis (Goode & Bean, 1879)	Gag grouper	0.0140	0.7054
**Seasonal**	**Before Control - After Control, Overall between-group dissimilarity = 0.4826**			
	Lagadon rhomboides (Linnaeus, 1766)	Pinfish	0.0674	0.1396
	Orthopristis chrysoptera (Linnaeus, 1766)	Pigfish	0.0539	0.2513
	Gerreidae spp. (Goode and Bean, 1879)	Mojarra spp.	0.0508	0.3567
	Leiostomus xanthurus (Lacepède, 1802)	Spot	0.0448	0.4496
	Paralichthys spp. (Jordan and Gilbert, 1882)	Flounder spp.	0.0347	0.5215
	Stephanolepis hispidus (Linnaeus, 1766)	Planehead filefish	0.0332	0.5903
	Bairdiella chrysoura (Lacepède, 1802)	Silver Perch	0.0240	0.6400
	Lutjanus griseus (Linneaus, 1758)	Grey Snapper	0.0220	0.6856
	Opsanus tau (Linnaeus 1766)	Toadfish	0.0176	0.7222
	Diplodus holbrookii (Bean, 1878)	Spottail pinfish	0.0158	0.7549
	**Before Hurricane - After Hurricane, Overall between-group dissimilarity = 0.5023**			
	Lagadon rhomboides (Linnaeus, 1766)	Pinfish	0.0636	0.1266
	Orthopristis chrysoptera (Linnaeus, 1766)	Pigfish	0.0610	0.2480
	Leiostomus xanthurus (Lacepède, 1802)	Spot	0.0473	0.3422
	Gerreidae spp. (Goode and Bean, 1879)	Mojarra spp.	0.0389	0.4197
	Paralichthys spp. (Jordan and Gilbert, 1882)	Flounder spp.	0.0376	0.4945
	Stephanolepis hispidus (Linnaeus, 1766)	Planehead filefish	0.0372	0.5685
	Bairdiella chrysoura (Lacepède, 1802)	Silver Perch	0.0306	0.6294
	Archosargus probatocephalus (Walbaum, 1792)	Sheepshead	0.0219	0.6731
	Diplodus holbrookii (Bean, 1878)	Spottail pinfish	0.0184	0.7098
	Lutjanus griseus (Linneaus, 1758)	Grey Snapper	0.0178	0.7452
	**Before Control - Before Hurricane, Overall between-group dissimilarity = 0.4432**			
	Lagadon rhomboides (Linnaeus, 1766)	Pinfish	0.0580	0.1309
	Orthopristis chrysoptera (Linnaeus, 1766)	Pigfish	0.0544	0.2537
	Leiostomus xanthurus (Lacepède, 1802)	Spot	0.0456	0.3566
	Stephanolepis hispidus (Linnaeus, 1766)	Planehead filefish	0.0358	0.4373
	Paralichthys spp. (Jordan and Gilbert, 1882)	Flounder spp.	0.0329	0.5114

	Gerreidae spp. (Goode and Bean, 1879)	Mojarra spp.	0.0242	0.5659
	Bairdiella chrysoura (Lacepède, 1802)	Silver Perch	0.0239	0.6198
	Diplodus holbrookii (Bean, 1878)	Spottail pinfish	0.0206	0.6663
	Sygnathus spp. (Jordan and Gilbert 1882)	Pipefish spp.	0.0183	0.7077
	Opsanus tau (Linnaeus 1766)	Toadfish	0.0180	0.7482
	**After Control - After Hurricane, Overall between-group dissimilarity = 0.4856**			
	Lagadon rhomboides (Linnaeus, 1766)	Pinfish	0.0596	0.1228
	Orthopristis chrysoptera (Linnaeus, 1766)	Pigfish	0.0543	0.2346
	Gerreidae spp. (Goode and Bean, 1879)	Mojarra spp.	0.0471	0.3316
	Leiostomus xanthurus (Lacepède, 1802)	Spot	0.0426	0.4194
	Paralichthys spp. (Jordan and Gilbert, 1882)	Flounder spp.	0.0412	0.5044
	Bairdiella chrysoura (Lacepède, 1802)	Silver Perch	0.0314	0.5691
	Lutjanus griseus (Linneaus, 1758)	Grey Snapper	0.0296	0.6301
	Stephanolepis hispidus (Linnaeus, 1766)	Planehead filefish	0.0285	0.6888
	Lutjanus synagris (Linneaus, 1758)	Lane Snapper	0.0188	0.7274
	Archosargus probatocephalus (Walbaum, 1792)	Sheepshead	0.0158	0.7599

Results of Similarity Percentages (SIMPER) analysis indicating the species that contribute the most to dissimilarity across mBACI groups based on Bray-Curtis dissimilarities calculated from fourth-root transformed abundance data. Only the top 10 species that contribute the most to between-group dissimilarities are listed.

S7 Table. Seasonal ANOVA and Tukey HSD for mBACI, Arthur- and Matthew BACIs

		ANOVA				Tukey HSD	
	**Response**	**Effect**	**DF**	**F**	**p**	**Comparison**	**p**
**mBACI**	CPUE $^{1/4}$ (km^{-1})	Period	1, 650	77.463	<0.001		
		Year Type	1, 650	2.983	0.085		
		Period*Year Type	1, 650	2.069	0.151		
	CPUE-Lr $^{1/4}$ (km^{-1})	Period	1, 650	59.551	<0.001	CB-IB	0.517
		Year Type	1, 650	0.001	0.976	CA-IA	0.120
		Period*Year Type	1, 650	6.802	0.009*	CB-CA & IB-IA	0.002, <0.001
						CB-IA, IB-CA	<0.001, <0.001
	Richness	Period	1, 650	0.484	0.487	CB-IB	0.155
		Year Type	1, 650	1.467	0.226	CA-IA	0.725
		Period*Year Type	1, 650	4.016	0.046*	CB-CA & IB-IA	0.798, 0.219
						CB-IA, IB-CA	0.987, 0.907
**Arthur BACI**	sqrt(CPUE) (km^{-1})	Period	1, 183	47.707	<0.001		
		Year Type	1, 183	4.473	0.0358		
		Period*Year Type	1, 183	0.255	0.614		
	sqrt(CPUE-Lr) (km^{-1})	Period	1, 183	70.995	<0.001		
		Year Type	1, 183	32.801	<0.001		
		Period*Year Type	1, 183	3.379	0.068		
	Richness	Period	1, 183	0.198	0.657		
		Year Type	1, 183	7.078	0.009		
		Period*Year Type	1, 183	0.418	0.519		
**Matthew BACI**	log(CPUE +1) (km^{-1})	Period	1, 139	50.137	<0.001	CB-IB	<0.001
		Year Type	1, 139	30.256	<0.001	CA-IA	<0.001
		Period*Year Type	1, 139	6.689	0.011*	CB-CA & IB-IA	0.102, <0.001
						CB-IA, IB-CA	0.880, <0.001
	log(CPUE-Lr +1) (km^{-1})	Period	1, 139	22.756	<0.001		
		Year Type	1, 139	25.473	<0.001		
		Period*Year Type	1, 139	0.828	0.364		
	sqrt(Richness) (km^{-1})	Period	1, 139	14.66	<0.001		
		Year Type	1, 139	21.04	<0.001		
		Period*Year Type	1, 139	2.25	0.136		

Summary results of seasonal-scale ANOVA tests across time period (before vs. after) and year type (control vs. impact year). Seasonal analyses include trawls conducted during the months of May-October. Response variables were transformed when necessary to meet assumptions of parametric statistics. Post-hoc test results are presented when the interaction term is significant at $p<0.05$. Abbreviations indicate treatments; CB = control before, IB = impact before, CA = control after, IA = impact after

S8 Table. GAM Model Summary Statistics for Seasonal mBACI

			edf	p	logLik	Dev	df.r	AIC	BIC
**Season**	**CPUE**	Control : Before	0.901	0.044	-2201.3	280.2	237.1	4409.1	4420.3
		Impact : Before	0.023	0.356	-2217.0	273.8	250.0	4438.1	4445.4
		Control : After	1.785	<0.001	-773.7	103.0	90.2	1555.4	1565.4
		Impact : After	1.708	<0.001	-846.4	119.5	104.3	1700.6	1711.0
	**CPUE-Lr**	Control : Before	0.637	0.129	-1703.6	278.3	237.4	3413.0	3423.3
		Impact : Before	0.003	0.426	-1817.2	290.9	250.0	3638.4	3645.5
		Control : After	1.672	<0.001	-582.9	99.4	90.3	1173.5	1183.2
		Impact : After	1.545	<0.001	-653.7	126.3	104.5	1314.8	1324.7
	**Richness**	Control : Before	<0.001	0.466	-539.6	218.1	238.0	1083.2	1090.2
		Impact : Before	1.755	<0.001	-559.8	199.1	248.2	1127.4	1141.0
		Control : After	1.437	0.001	-220.1	89.9	90.6	447.8	457.3
		Impact : After	1.711	<0.001	-241.0	106.7	104.3	489.8	500.1

All GAM models were run for the seasonal time frame against days since storm as the independent variable and built using a cubic regression spline with penalized shrinkage, a maximum of three degrees of freedoms, negative binomial error distribution with log link function, and restricted maximum likelihood smoothing parameter. edf = effective degrees of freedom, logLik = log likelihood, Dev = deviance, df.r = residual degrees of freedom, AIC = Akaike information criterion BIC = Bayesian information criterion.

S9 Table. Hurricane Arthur GLM Summary Statistics

			Term	Est.	z-value	P-value	df.r	AIC	BIC
Arthur	CPUE	Control : Before	Intercept	6.504	21.033	<0.001	21	330	333
			Period*Year Type	0.010	1.021	0.307			
		Impact : Before	Intercept	5.798	17.812	<0.001	49	763	769
			Period*Year Type	-0.022	-2.414	0.016			
		Control : After	Intercept	6.081	18.135	<0.001	41	561	566
			Period*Year Type	-0.0.10	-2.131	0.033			
		Impact : After	Intercept	6.558	42.047	<0.001	68	919.3	926.1
			Period*Year Type	-0.020	-8.100	<0.001			
	CPUE-Lr	Control : Before	Intercept	3.999	9.768	<0.001	21	240	243
			Period*Year Type	-0.004	-0.311	0.756			
		Impact : Before	Intercept	4.358	13.614	<0.001	49	597	603
			Period*Year Type	-0.017	13.872	0.060			
		Control : After	Intercept	3.874	13.872	<0.001	41	367	372
			Period*Year Type	-0.012	-2.814	0.005			
		Impact : After	Intercept	4.650	30.441	<0.001	68	681	687
			Period*Year Type	-0.016	-6.520	<0.001			
	Richness	Control : Before	Intercept	2.149	11.904	<0.001	21	104	107
			Period*Year Type	0.009	1.564	0.118			
		Impact : Before	Intercept	2.126	12.479	<0.001	49	236	242
			Period*Year Type	0.004	0.735	0.463			
		Control : After	Intercept	1.959	14.332	<0.001	41	217	222
			Period*Year Type	-0.002	-0.750	0.454			
		Impact : After	Intercept	2.067	25.078	<0.001	68	371	377
			Period*Year Type	<0.001	0.029	0.977			

Negative binomial generalized linear models for seasonal-scale trend analysis of Hurricane Arthur (2014) versus 2015 as the control year. Est = estimate, df.r = residual degrees of freedom, AIC = Akaike information criterion BIC = Bayesian information criterion.

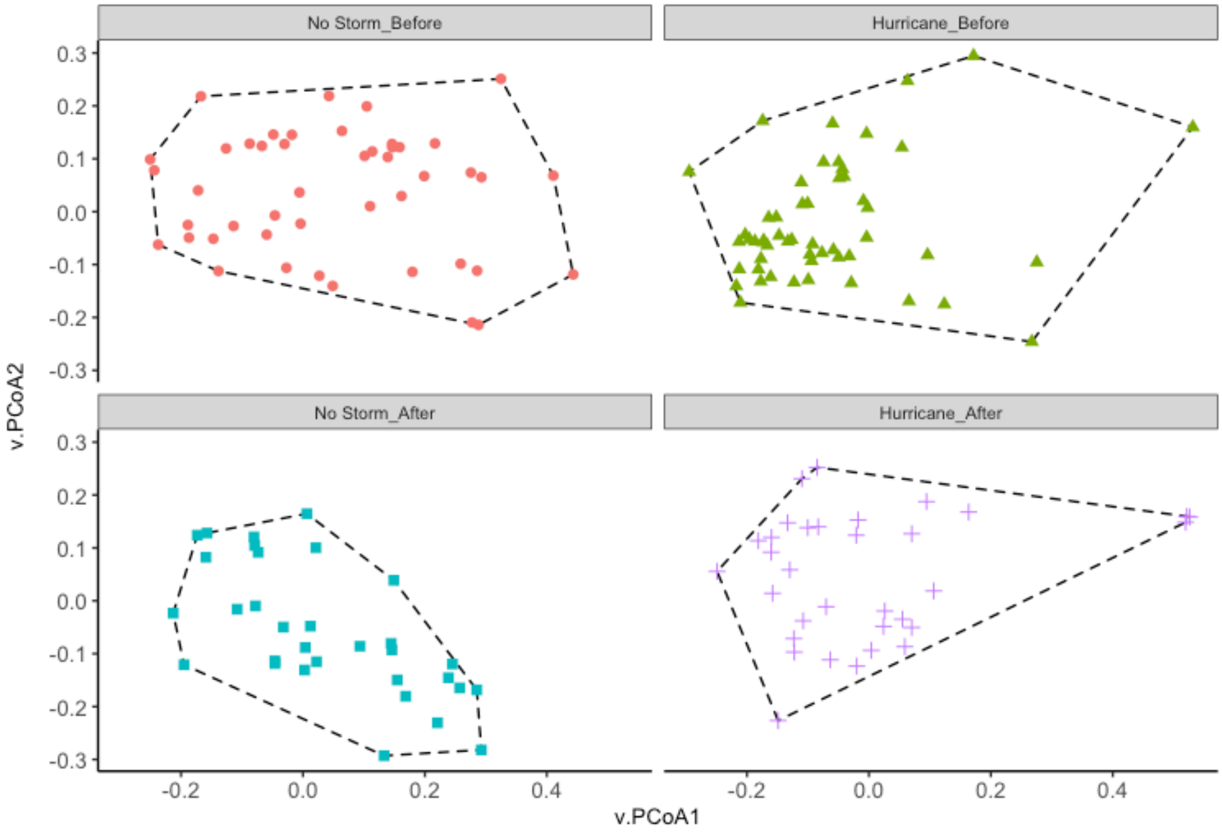

S2 Figure. Individual mBACI treatment PCoA ordinations of short-term communities
This figure demonstrates the potential difference/lack of difference in short-term community dispersion within each mBACI treatment using Principle Coordinates Analysis. Convex hulls are drawn in dashed lines through the outer-must points.

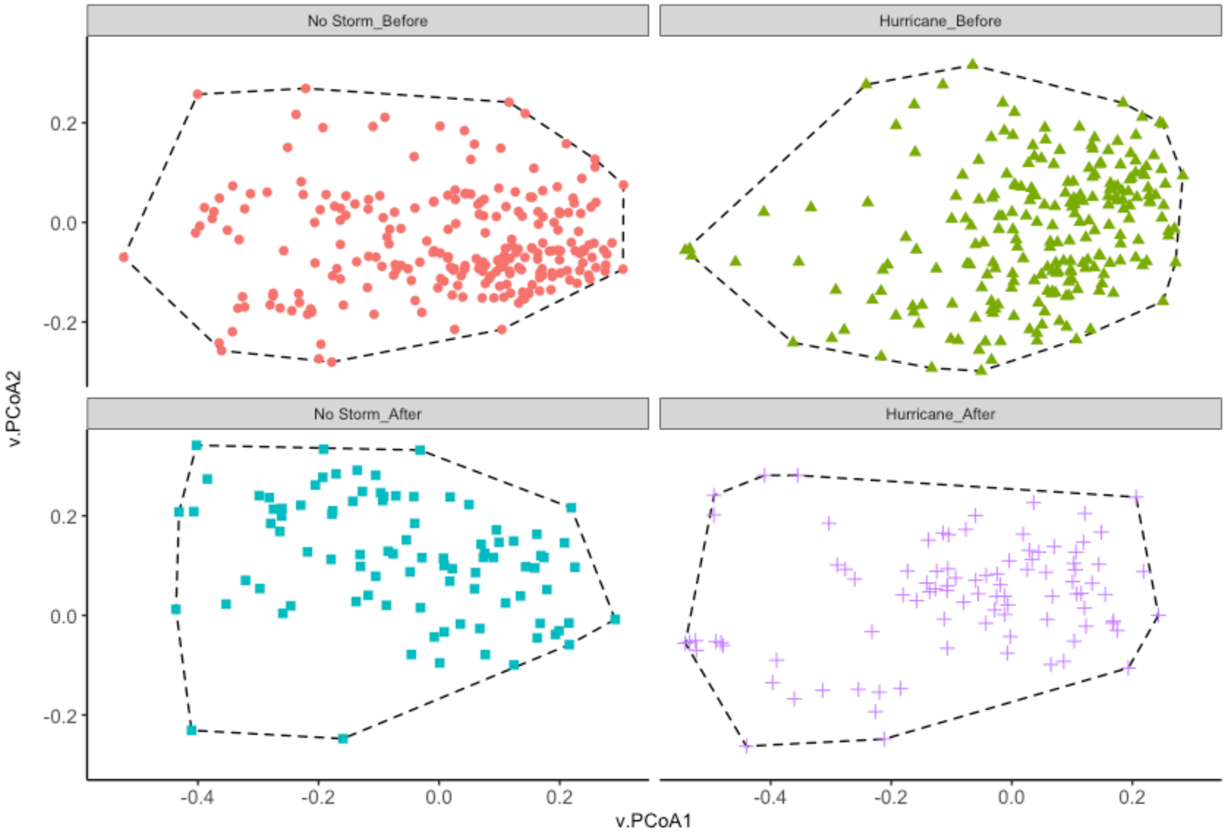

S3 Figure. Individual mBACI group PCoA ordinations of seasonal communities
This figure demonstrates the potential difference/lack of difference in seasonal community dispersion within each mBACI treatment using Principle Coordinates Analysis. Convex hulls are drawn in dashed lines through the outer-must points.

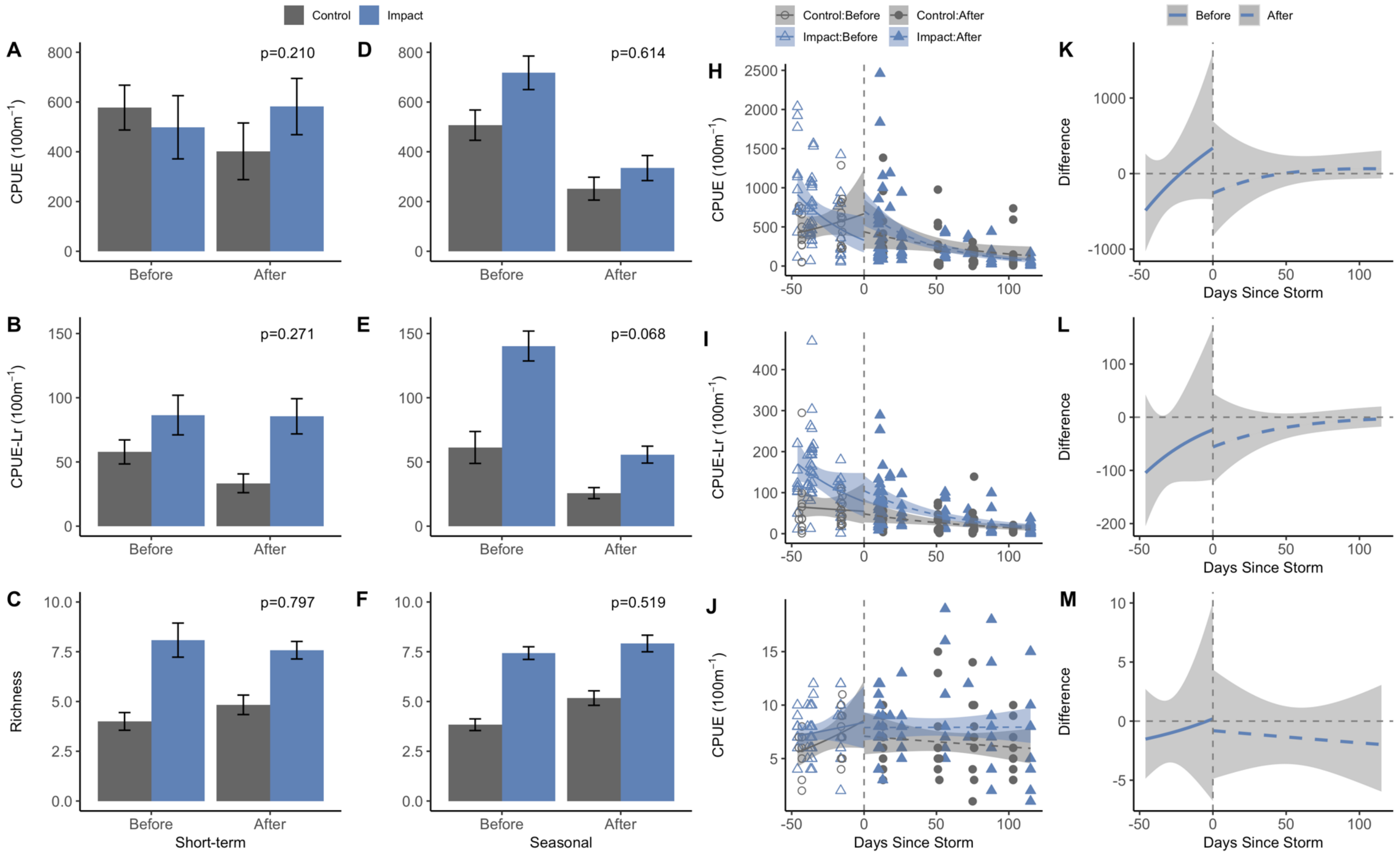

S4 Figure. Short-term and seasonal fish catches and species richness across time periods and year type for Hurricane Arthur (July 2014) compared to 2015 (control year). Only means are presented for short-term comparisons (column 1); whereas means, trend, and difference between control and impact trends (columns 2-4, respectively) are depicted for seasonal comparisons. Catch per unit effort (CPUE) is presented in row 1; CPUE calculated sans *L. rhomboides* is presented in row 2, and species richness is row 3. P-values indicate the significance of the interactive ANOVA term. Error bars represent standard error. Smoothed lines represent generalized additive models (y ~ s(Days to Storm), k = 3) for both hurricane and storm-free years based on a cubic regression spline with shrinkage and 95% confidence intervals.

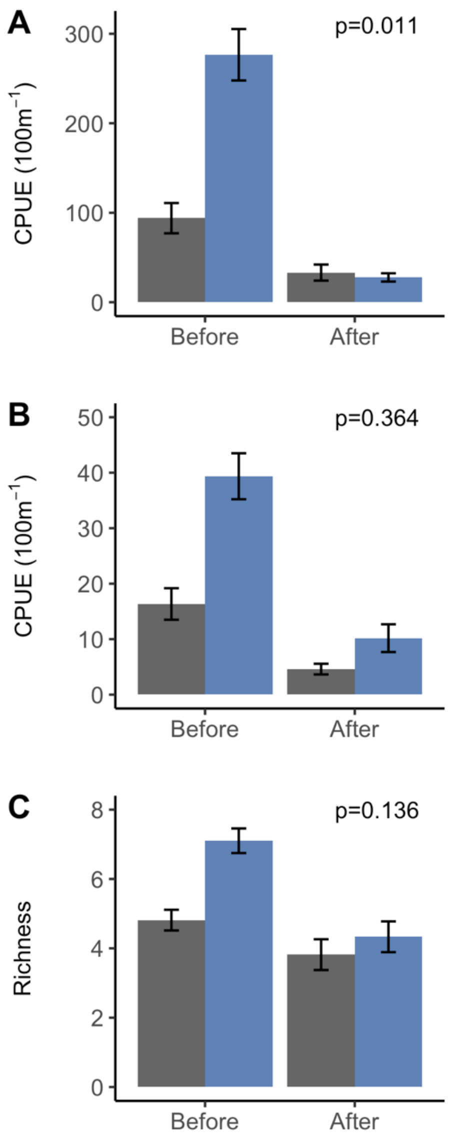

S5 Figure. Seasonal fish catch per unit effort (A), catch per unit effort sans *Lagodon rhomboides* (B) and species richness (C) across time periods (before vs. after) and year type (control vs. impact) for Hurricane Matthew (October 2016) compared to 2017 (control year). Only means are presented for seasonal comparisons (column 1), as the closest trawl samples prior to hurricane Matthew occurred outside of the short-term window and all trawls that occurred after the storm were conducted on the same day. P-values indicate the significance of the interactive ANOVA term. Error bars represent standard error.
